# Characterization of the past and current duplication activities in the human 22q11.2 region

**DOI:** 10.1186/1471-2164-12-71

**Published:** 2011-01-26

**Authors:** Xingyi Guo, Laina Freyer, Bernice Morrow, Deyou Zheng

**Affiliations:** 1 Department of Neurology, Albert Einstein College of Medicine, Bronx, NY 10461, USA; 2 Department of Genetics, Albert Einstein College of Medicine, Bronx, NY 10461, USA; 3 Department of Neuroscience, Albert Einstein College of Medicine, Bronx, NY 10461, USA

## Abstract

**Background:**

Segmental duplications (SDs) on 22q11.2 (LCR22), serve as substrates for meiotic non-allelic homologous recombination (NAHR) events resulting in several clinically significant genomic disorders.

**Results:**

To understand the duplication activity leading to the complicated SD structure of this region, we have applied the A-Bruijn graph algorithm to decompose the 22q11.2 SDs to 523 fundamental duplication sequences, termed subunits. Cross-species syntenic analysis of primate genomes demonstrates that many of these LCR22 subunits emerged very recently, especially those implicated in human genomic disorders. Some subunits have expanded more actively than others, and young *Alu *SINEs, are associated much more frequently with duplicated sequences that have undergone active expansion, confirming their role in mediating recombination events. Many copy number variations (CNVs) exist on 22q11.2, some flanked by SDs. Interestingly, two chromosome breakpoints for 13 CNVs (mean length 65 kb) are located in paralogous subunits, providing direct evidence that SD subunits could contribute to CNV formation. Sequence analysis of PACs or BACs identified extra CNVs, specifically, 10 insertions and 18 deletions within 22q11.2; four were more than 10 kb in size and most contained young *AluY*s at their breakpoints.

**Conclusions:**

Our study indicates that *AluY*s are implicated in the past and current duplication events, and moreover suggests that DNA rearrangements in 22q11.2 genomic disorders perhaps do not occur randomly but involve both actively expanded duplication subunits and *Alu *elements.

## Background

Segmental duplications (SDs) or low copy repeats (LCRs), defined as continuous non-repetitive DNA sequences that are found at two or more genomic locations, comprise ~5% of the human genome [1-3]. SDs can mediate meiotic unequal non-allelic homologous recombination (NAHR) events, resulting in genomic rearrangement and sometimes altered gene dosage within the intervening regions such as those on 22q11.2. Segmental duplications or low copy repeats on 22q11.2 (often referred to as LCR22s) [4,5] are of great interest because they have been associated with four different human disorders [6]: velo-cardio-facial/DiGeorge syndrome (VCFS/DGS) (MIM #192349 [7] or MIM #188400 [8,9]), the reciprocal duplication syndrome [5,10], der(22) syndrome [11], and cat-eye syndrome [12]. Despite extensive molecular studies in the past decade, the precise position of the breakpoints within the two LCR22s associated with most of these syndromes, LCR22-2 and -4, remain largely elusive. This is due to their high sequence similarity (97%-99%) and that there are eight related LCR22 blocks on 22q11.2, comprising over 11% of the region, making it difficult to identify paralogous sequences unique to one or the others [5,13,14]. Interestingly, characterization of the genomic sequence within and near LCR22s demonstrated that both *Alu *repeat elements and AT-rich repeats were enriched and likely involved in many of the past unequal crossover duplications that have shuffled DNAs among blocks and given rise to the current complex genomic architecture of LCR22s [14]. This is consistent with findings from genome-wide analyses of human SDs [15,16].

**Figure 1:**
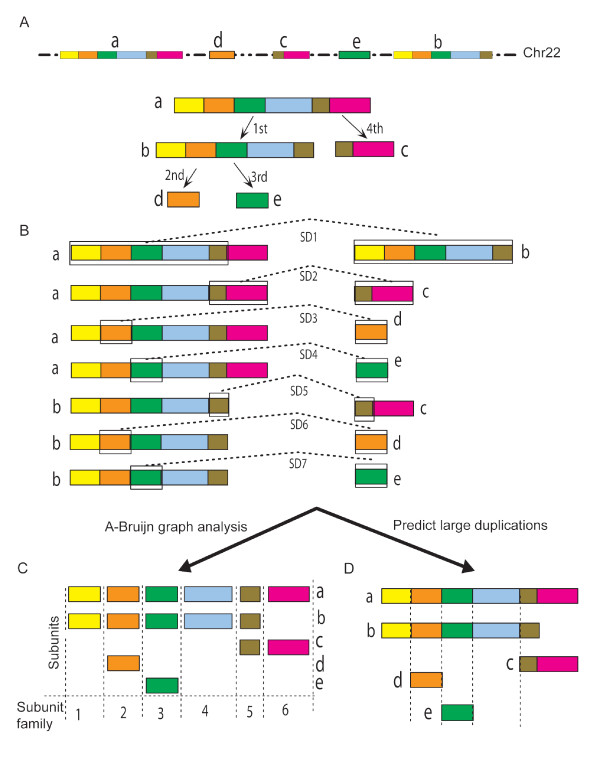
**A schematic cartoon for the decomposition of segmental duplications into duplication subunits and the construction of map for putative duplication events**. (A) Five hypothetical duplication loci (a-e) are depicted with their duplication history shown below. Note that in real cases the historical duplication directions can only be inferred as duplications occurred in the past and are actually invisible. (B) The segmental duplication data for these five loci are represented by seven pairs of duplicons (boxes connected by dash lines). A total of 202 such pairs exist for 22q11.2 based on sequence comparison. (C) Fifteen duplication subunits (forming six paralogous families) decomposed from the pair-wise alignment information in B. (D) The five duplication loci are grouped and all loci are then aligned to the "a" locus, which is the largest one. Note that the entire locus "a" has to be derived from the merge of left duplicons in SD1 and SD2. 33 such duplication groups were defined for 22q11.2, containing 174 duplication loci (see Figure 2B).

To understand the duplication architecture of LCR22 and more importantly to gain insight to the molecular mechanism behind high incidence of pathogenic LCR22 rearrangements causing human congenital malformation syndromes, we have applied A-Bruijn graph algorithm to decompose the LCR22 architecture to fundamental duplication subunits at nucleotide-level resolution, without any bias to either genes or pseudogenes as was in the case of previous studies focused on LCR22s that are mentioned above (Figure 1). Our study moreover found unexpectedly high genetic variation between and within SDs, indicating them as highly dynamic in the genome. This is supported by the fact that many subunit copies emerged recently, at either the human or the African great ape lineage, through both small and large-scale duplications. Highly active subunits were found with significant enrichment of *AluY*, a young short interspersed nuclear element (SINE), at their ends or short adjacent sequences. This repeat and some subunits associated with it, continue to actively mediate CNV generation in human LCR22 region. These CNVs could alter risk to genomic disorders on 22q11.2 by making them better or worse substrates for meiotic recombination.

## Results

### Segmental duplications on 22q11.2 and their gene content

Segmental duplications (SDs) or low copy repeats directly contribute to the genome dynamics and meiotic instability of human 22q11.2. In this study, we began by surveying the content and extent of duplications within the 22q11.2 region using SDs with sequence identity ≥ 90% and length ≥ 1 kb that have been annotated by Dr. Eichler's group through Whole Genome Assembly Comparison (WGAC) and Whole Genome Shotgun Sequences Detection (WSSD) [1,2]. In total, 202 pairs of SD sequences (often termed duplicons) were located on 22q11.2 (chr22:17,000,000-24,000,000, NCBI36/hg18), accounting for ~1.8 Mb (26%) of its bases. The rest of the DNA sequence between SDs was referred here as "unique sequences" (the corresponding locations thereby as "unique regions" as they were not explicitly involved in duplications within this region). The sequence divergence of the SDs on 22q11.2 was relatively low, with a median of 3.6%, and with 58% of them diverging < 4% (Additional file 1, Figure S1). By comparison, the average sequence divergence for all human SDs was 6% while 25% of them diverged < 4%. The average length of SDs in 22q11.2 was 13 kb (ranging from 1 to 162 kb), while a significant negative correlation between length and divergence was observed for these SDs (r = -0.46, p < 6 × 10^-12^; Additional file 1, Figure S1), suggesting that either many old duplicated sequences have experienced significant nucleotide loss after their emergence or recent duplication events produced mainly large SDs.

**Figure 2:**
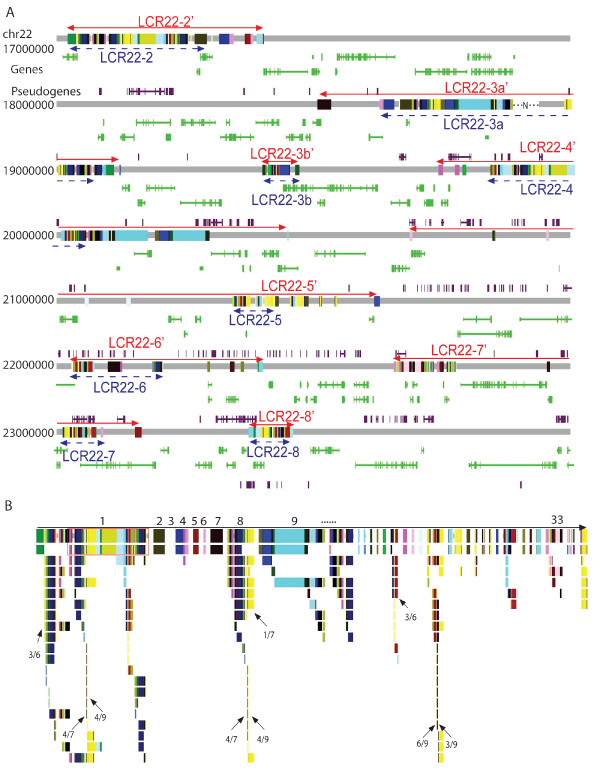
**The mosaic architecture of segmental duplications in the human 22q11.2 region**. (A) Duplicated subunits, genes and pseudogenes. The 22q11.2 region is depicted as a grey line and colored boxes for unique and SD sequences, respectively. Eight duplicated blocks are labelled with red arrow lines for current boundary definition (Table 1) and blue arrow lines for the previous definition [14]. Paralogous subunits (i.e., in the same subunit family) are shown with same color. For simplification, both genes (green) and pseudogenes (purple) were drawn without names. (B) Hierarchy of non-overlapping duplicated loci. A total of 33 groups of duplication loci in 22q11.2 were identified and all loci were aligned to the largest locus of their corresponding groups (all subunits have the same color as in Figure 2A). Horizontal order shows relative chromosome locations with white spaces added to separate sequences in distinct duplication groups. Arrows point to paralogous subunits at the breakpoints of recurrent (> 5) duplications; numbers below them are the total subunits at breakpoints and subsets with Alu elements. A gap in LCR22-3a' was represented by a dash line with 'N'.

It is well known that DNA duplications can lead to new copies of genes or create pseudogenes [17,18]; and both genes [1] and pseudogenes [19] are enriched within SDs. Using the most recent and comprehensive data from the ENCODE gene annotation group [20], we have surveyed the pseudogene and gene content on 22q11.2 (Figure 2A). Overall, the SD regions of 22q11.2 were enriched with about three times more pseudogenes than non-SD regions, as 6% and 2% of the base pairs in the two regions were annotated as pseudogenic, respectively (p < 0.001). The percentages of base pairs corresponding to coding exons, however, were more similar (6% in SD *vs *8% in the unique regions). These numbers are higher than average for the human genome, which reflect the known enrichment of genes in 22q11.2.

### Decomposition of 22q11.2 SDs to duplication subunits and blocks

To establish a reference frame for studying intra-LCR22 duplication activity and to gain insight to their disease susceptibility, we decided to decompose SDs to basic duplication subunits, which represent the smallest continuous sequences that have been involved in at least one independent intra-LCR22 duplication event. As illustrated in Figure 1, the boundaries of subunits denote potential breakpoints, although some of which may be implicated in more duplications than others. Indeed, those recurrent subunits (e.g., subunits in orange and green in Figure 1) constitute core duplicons that have given rise to the majority of human SDs [21]. Applying the program, RepeatGluer that utilized A-Bruijn graphs to resolve mosaic structures of repeat sequences [22], we have decomposed the 202 pairs of SDs in 22q11.2 to 523 non-overlapping subunits (Figure 2; Additional file 2, Table S1). These subunits ranged from 30 bp to 63.8 kb, with a mean length of 3,240 bp (median is 1,333 bp). Previously, Jiang *et al *have applied the A-Bruijn graph algorithm to decompose the human SDs to subunits [21,23] and the resulting subunits were defined very similar to current ones (see Methods). With previous definition of blocks as guidance [24], we further partitioned the 523 subunits into eight duplication blocks, LCR22-2', LCR22-3a', LCR22-3b', and LCR22-4' to LCR22-8' (Table 1 and Figure 2A; the " ' " denotes a larger interval than the classic LCR22s, delineated previously with LCR22-2 and LCR22-4 as the base reference for defining duplications [14,25]). In comparison with the past definition, currently defined blocks were typically longer and encompassed more SDs. Our current analysis has particularly expanded the duplicated content of LCR22-4' and LCR22-7' with newly described SDs (the distal part of LCR22-4' and the proximal part of LCR22-7' in Figure 2). As shown with paralogous relationship (depicted by same colors) in Figure 2, the newly incorporated SD subunits are surely components of the evolutionary products of the past DNA duplications in 22q11.2, and thus should be added to the "classic" definition of LCR22s and included for future study. Additionally, the delineation of SDs to individual subunits shows the mosaic evolutionary relationship within LCR22s much more clearly.

**Table 1:** Summary of LCR22 blocks and their subunits

Block Name	Start	End	Number of Duplicated Subunits	Mean Size (deviation) of Subunits	Subunits present only in Human (%)	Subunits in Chimp (%)	Subunits in Orangutan (%)	Subunits in Macaque (%)
LCR22-2'	17022491	17403012	77	3872 (623)	20.8	62.3	54.6	32.5

LCR22-3a'	18508560	19074436	80	4720 (944)	38.8	36.3	47.5	7.5

LCR22-3b'	19351885	19422337	16	3226 (1183)	18.8	81.3	25.0	25.0

LCR22-4'	19694655	20401391	82	5420 (1162)	25.6	65.9	37.8	12.2

LCR22-5'	20638869	21579072	59	2593 (305)	5.1	91.5	71.2	59.3

LCR22-6'	21979113	22351303	33	3473 (765)	3.0	78.8	84.9	87.9

LCR22-7'	22607349	23115227	148	1171 (174)	16.9	80.4	73.7	10.8

LCR22-8'	23324435	23410013	28	2942 (590)	3.6	78.6	92.9	46.4

As shown in Figure 2, the eight LCR22' blocks are separated and flanked by unique (i.e., non-SD) sequences. The clustering of subunits in these blocks suggests that some sequence features may have rendered them as targets of duplication "hotspots". The shared similarity at macro-scale in their subunit arrangements among LCR22-2', LCR22-3a' and LCR22-4' confirms that these three blocks may arise from few instances of large-scale duplication events involving many adjacent subunits simultaneously [26,27]. Note that part of LCR22-3a' remains a missing gap in the reference human genome sequence. In contrast, LCR22-3b', LCR22-5', LCR22-6', LCR22-7', and LCR22-8' display a different architectural pattern consisting mostly of small subunits connected in a discrete manner. For example, the average size of subunits in LCR22-4' is 5,420 bp, which is approximately twice as large as that of LCR22-5' (2,593 bp) and four times larger than that of LCR22-7' (1,171 bp). Analysis of the relative abundance of large subunits, defined by either >5 kb, >10 kb or >20 kb (data not shown), also showed that large subunits were predominantly located in LCR22-2', LCR22-3a' and LCR22-4'. Since large SDs overall show low sequence divergence as described above (Additional file 1, Figure S1), this result further suggests that the bulk DNAs constituting these three blocks was likely generated more recently during evolution than other LCR22 blocks. The sporadic presence of a few small subunits specifically in each of the three "young" blocks indicates that new micro-scale duplications have occurred after their initial formation.

### Occurrence of LCR22s in other primate genomes

As mentioned above, the majority of SDs on 22q11.2 shows < 4% sequence divergence. Accordingly, we estimated that the majority (>58%) of duplicated sequences emerged between 10-20 million years ago and after the divergence of human and macaque lineages, based on an estimation of 3% divergence between duplicated sequences per 10 million years [16]. To further explore the evolutionary history of these SDs, we have surveyed and characterized the syntenic regions of human LCR22' sequences in the chimpanzee, orangutan, and macaque genomes.

**Figure 3:**
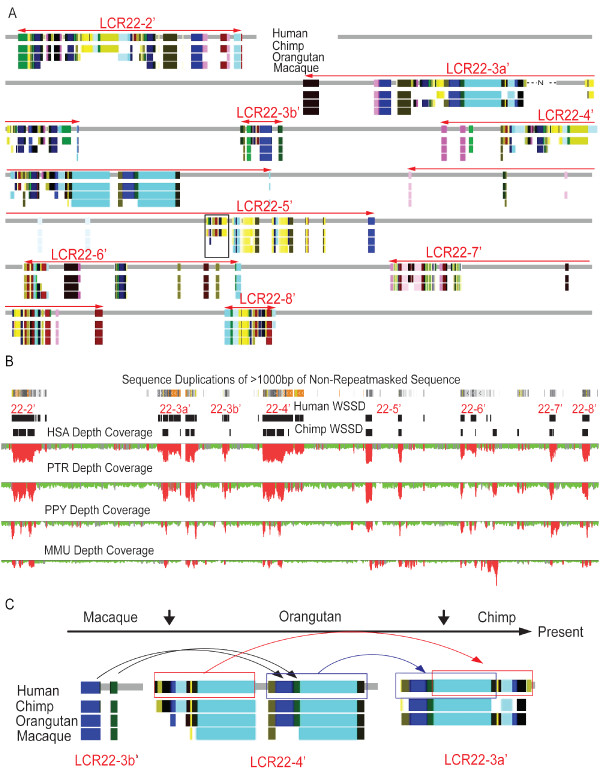
**Synteny of SDs on 22q11.2**. (A) The syntenic relationship of the subunits with chimpanzee, orangutan and macaque is shown as present (matching color boxes) or absent (white). This map was derived from our analysis of the multi-genome alignment data in the Ensembl database (see Methods). The boxed region in LCR22-5' was subsequently confirmed by PCR to be absent in the macaque genome (see Additional file 3, Figure S2). (B) Comparison of primate segmental duplications. The data were retrieved from a previous study using WSSD analysis for SD detection [29]. The depth of sequence read coverage (number of shot-gun sequencing reads in 5-kb windows) is depicted for human (HAS), chimpanzee (PTR), orangutan (PPY) and macaque (MMU) based on alignment of reads against the human genome. Putative duplicated regions with excess read depth (more than three standard deviation of the mean) are shown in red with unique regions in green. Human and chimp SDs derived from depth analysis are also shown below the human SDs derived from WGAC analysis (top). The data here suggest that most of the sequences in LCR22-2', -3a' and -4' are shared between human and chimpanzee and their duplications likely occurred after the split of the African great apes from Asian great apes. Interestingly, the human-specific SDs in LCR22-3a' and -4' show higher sequence identity (represented by light to dark orange color) than the rest of the SDs (light to dark grey). (C) Past duplication events that may have generated the homology between LCR22-3a' and LCR22-4'. Arrow lines represent putative duplication directions. The large cyan subunit in LCR22-3a' may have arisen from either the proximal or distal paralogous sequences in LCR22-4'.

Using multiple genome alignment data from the Ensembl database [28] and with extra filtering to improve syntenic map for duplicated sequences (see Methods for details), we found that 70%, 61%, and 26% of the 22q11.2 SD subunits had unambiguous syntenic sequences in chimpanzee, orangutan, and macaque (Table 1), respectively, and all together 81% of subunits had syntenic sequences detected in at least one of the three current assembles of non-human primate genomes (Figure 3A). More specifically, 39%, 21%, and 26% of duplicated subunits in LCR22-3a', LCR22-2', and LCR22-4', respectively, appeared specific to the human genome (Table 1; Figure 3A). By comparison, only 3% to 19% of the subunits in LCR22-3b', LCR22-5', 6', 7', and 8' exhibited human specificity. We have also carried out a PCR assay to support our syntenic analysis (Additional file 3). Together with the results from our analysis of subunit distribution in 22q11.2 (above), our cross-species syntenic analysis demonstrates that most sequences in LCR22-2', LCR22-3a', and LCR22-4' were generated from more recent duplication events.

This is also supported firstly by FISH mapping experiments using probes to four well-characterized genes in the LCR22 region that detected signals for the presence of LCR22-6, LCR22-7, LCR22-8 sequences in chimpanzee, orangutan and macaque [24]. Secondly, phylogenetic analysis of *BCR, GGT, GGTLA *and *USP18 *genes or pseudogenes in human LCR22s also indicated LCR22-2, LCR22-3a and LCR22-4 were evolutionarily close but they were distant from the other LCR22 blocks [24]. Thirdly, previous comparative analysis of SDs in four primate genomes indicated in particular that those located in the distal halves of LCR22-2 and LCR22-4, were generated more recently (Figure 3B). This important finding is probably relevant to the fact that most of the genomic disorders on 22q11.2 are mapped to LCR22-2, LCR22-3a and LCR22-4 regions, although ascertainment due to deletion of critical dosage sensitive genes associated with known syndromes provides significant bias.

### Re-construction of large duplication events

One of the main goals of our study is to identify and characterize subunits that are implicated in frequent duplications, as such subunits may host recombination "hotspots" of genomic disorders in 22q11.2. We started by grouping subunits into paralogous families (see hypothetical example in Figure 1) to capture their putative intra-LCR22 duplication relationships. A total of 122 subunit families were assembled from the 523 subunits (Additional file 2, Table S1). The sizes of these families range from 2 to 16 subunits, with one third of these families having fewer than six members.

As shown with cartoons in Figure 1, both SD duplicons and subunits need to be appropriately merged and aligned in order to identify past duplication events from multiple candidates correctly. As such, we first merged physically overlapping SD duplicons to obtain duplication loci [see Methods for details, a duplication locus here was defined as a genomic region containing one or more (overlapping) duplicons not disrupted by unique sequence]. We determined a total of 147 duplication loci from the 22q11.2 SDs, and they were further separated to 33 groups based on sharing of paralogous subunits (Figure 2B). Finally, aligning all duplication loci to the largest locus of their respective group, as illustrated in Figure 1D, yielded a hierarchical structure representing putative duplication relationship of all SDs in 22q11.2 (Figure 2B). At the top first level, 33 distinct duplication loci were identified (top row in Figure 2B) and they accounted for 47% of all SD sequences in 22q11.2, suggesting that the remaining 53% SD sequences might have arisen from these 33 loci.

One feature emerging from the data in Figure 2B is that some subunit families are frequently located at the ends (i.e., breakpoints) of putative duplications, suggesting that they might have been highly active in mediating past duplication events. For example, at least six duplication events might have been mediated by subunits in the family that included a member at the 5' end of LCR22-2' (most left arrow in Figure 2B). Somewhat surprisingly, analysis of such subunit families significantly enriched at the boundaries of duplications (arrows in Figure 2B) revealed that 46% of the subunits implicated in frequent past duplication events harbored or were adjacent to *Alu *elements.

As shown in Figure 2B, most of the putative duplications in 22q11.2 involved relatively small (<10 Kb) duplicons and thus were not further pursued due to the limitation of our approach in resolving the donor and acceptor of a duplication event, but at least three duplication events operated on large duplicons (boxes in Figure 2B). The largest one (involving 40 subunits and containing 162 kb sequence) occurred between LCR22-2' and LCR22-4' (first box in Figure 2B). The other two large-scale duplications involved the largest subunit (~64 kb, second box in Figure 2); one occurred between LCR22-3a' and LCR22-4', and the other at the distal half of LCR22-4' (Figure 3C).

Further syntenic analysis (described above) showed that the duplication between LCR22-3a' and LCR22-4' occurred after the split of the macaque lineage, while the duplication at LCR22-4' might have occurred earlier (Figure 3A, C), although a previous complementary analysis comparing SDs in four primate genomes [29] indicated that both duplications occurred in the ancestral lineage of human and chimpanzee (Figure 3B). Future experiments, such as FISH mapping, are needed to resolve these two different findings. Interestingly, it appears that two independent duplications had inserted two subunits (blue and green subunits in Figure 3C) in front of the distal cyan subunit of LCR22-4' before the resulting sequence was then duplicated to LCR22-3a'. Alternatively, some subunits in LCR22-3a' may have originated from the proximal part of LCR22-4'. This uncertainty could not be resolved from sequence similarity, as the pair-wise sequence identity from the duplication events marked by the blue and red arrow is 99.5% and 99.6%, respectively, highlighting the challenge in reconstructing past duplication events accurately.

### Repeat elements and duplications on 22q11.2

**Figure 4:**
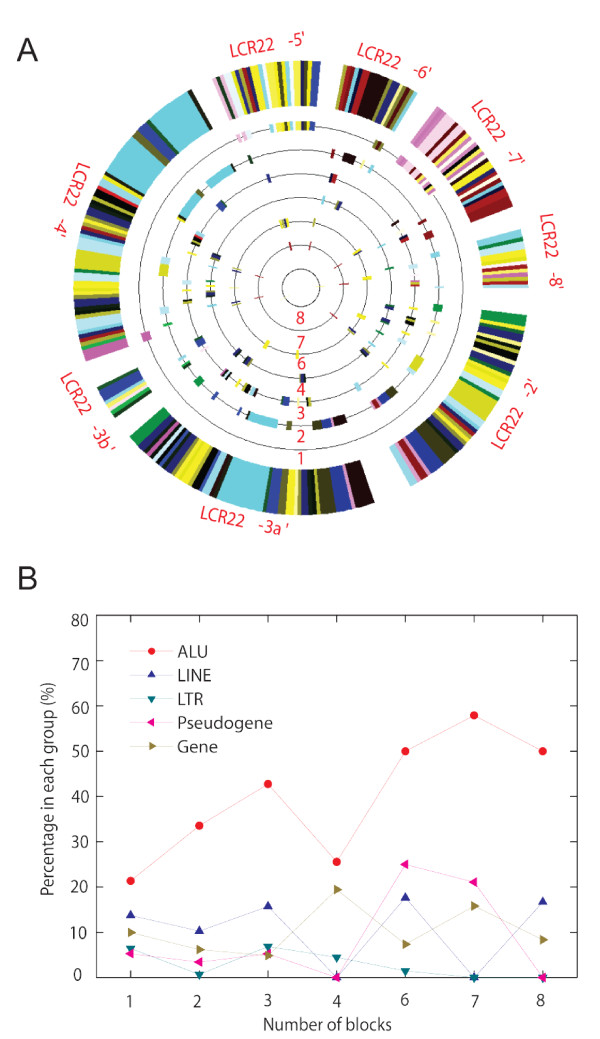
**Subunit family spreading in multiple LCR22' blocks is often adjacent to *Alu *repeats**. (A) The SD subunits were assigned to different layers of circles, whereas the numbers represent the total blocks in which a subunit family has one or more members. For example, a subunit family is given 3 if its members are found in 3 of the 8 blocks, and consequently all subunits of this family will be drawn in the circle labeled with "3". (B) Relationship between selected sequence features and block occupancy for SD subunits. The *x*-axis describes the number of blocks a subunit family occupies (A). The *y*-axis shows the percentage of subunit endpoints with a given sequence feature. No subunit family was found in and only in five blocks.

As shown in Figure 2, most duplication events involved in short sequences. We thus decided to investigate to what degree such small duplications have contributed to the mosaic duplication patterns of SDs in 22q11.2 and sequence shuffling between LCR22 blocks. The majority of subunit families (69%) had paralogs presence in two to four blocks, which seemed to be mostly a result of recent inter-block duplication among LCR22-2', LCR22-3' and LCR22-4' (Figure 4A). The subunit families in LCR22-2', -3', -4', -6' and -8' were quite active as more than 95% of their subunits had paralogs in other blocks. In contrast, 70% and 80% of the subunits in LCR22-5' and LCR22-7', respectively, existed as intra-block duplications (Figure 4A). Syntenic sequences to the tandem subunits of LCR22-7' were found in the genomes of chimpanzee and orangutan but not macaque (Figure 3), indicating the underlying duplication events occurred ~25 million years ago. Interestingly, 39 families occupied only in a single block, indicating their expansion was largely a result of local and potentially tandem duplications.

To search for potential sequence features mediating active duplications, we characterized the short sequences immediately adjacent (±10 bp) to duplicated subunits in 22q11.2. First, we calculated the abundances of different sequence features, e.g., *Alu*, *LINE-1*, gene, and pseudogene, and correlated them with the "duplication activity" of individual subunit families, measured by the number of blocks a family resided. The result showed that *Alu*/SINE elements were associated much more frequently with subunit families that have undergone active expansion (Figure 4B), suggesting *Alu*-mediated duplications might be responsible for most of the inter-block duplications. Interestingly, direct survey of the adjacent sequences of all 22q11.2 SD subunits also found that *Alu *was the most prevalent repeat, present in 32% of the 523 subunits. Of all the 339 *Alu *repeats next to the subunits, 30% of them were *AluSx *and 28% were *AluY*, followed by *AluJo *(11%), *AluSq *(8%), and *AluSg *(6%). A random simulation indicated that the associations of subunits with each of these different *Alu *types were significantly more than expected (p < 0.001). These findings are consistent with previous reports that *Alu*-mediated recombination events actively shuffled genes within LCR22 blocks and that young *Alu *elements (*AluY *and *AluS*) were frequently enriched at the end of SDs [15,30].

### CNVs flanked by paralogous subunits

**Figure 5:**
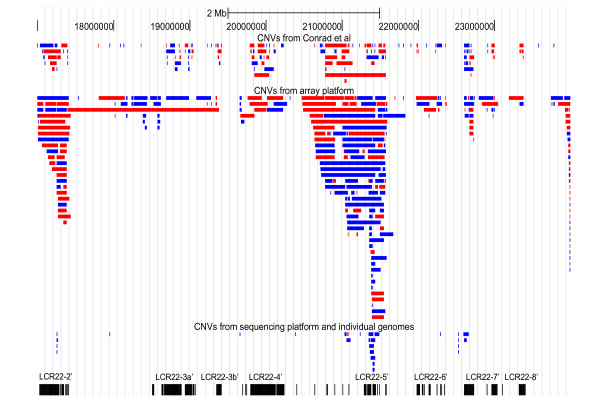
**Distribution of previously annotated CNVs in the 22q11.2 region**. The gain and loss CNVs collected from previous publications are shown with blue and red, respectively. The bottom row illustrates SD subunits. The figure was prepared using the UCSC browser.

SDs are a major source of genome instability and they have been suggested to play an important role in the etiology of copy number variations (CNVs), but the extent and features of CNVs in 22q11.2 have not been characterized to date. To explore this, we obtained all previously annotated human CNVs and overlaid them on our map of 22q11.2 SD subunits. Due to the high sequence identity of 22q11.2 SDs, which may cause cross-hybridization signals, we only considered genome-wide CNVs detected using relatively high-resolution technology (either using microarray with short probes or based on direct sequencing). We collected 452 CNVs from previous studies (69 gains and 72 losses > 460 bp [31]; 133 gains and 178 losses > 1 kb [32]; and other CNVs obtained from the Database of Genome Variants based on either paired-end fosmid clone mapping or individual personal genomes) (Figure 5). Respectively, these CNVs were enriched in SDs by two-fold *vs *non-SD region of 22q11.2. Further examination found that the two breakpoints for 13 of these CNVs (mean length 65 kb) were located to the same duplication subunit family (i.e., paralogous subunits), providing direct evidence that some pairs of paralogous subunits could indeed mediate CNV formation by NAHR (Table 2). Interestingly, 12 of these 13 CNVs were deletions. More surprisingly, except one in LCR22-2', all SD-overlapping CNVs were located to either LCR22-5' or LCR22-7' regions (Figure 5), where paralogous subunits frequently exist in tandem.

**Table 2:** Total of 13 previously detected CNVs (from the Database of Genomic Variants) with breakpoints located to the paralogous subunits (see Figure 7A)

Index	CNV start	CNV End	Length (bp)	Gain/Loss	Left subunit	Right subunit	Subunit family ID	Block	Technology
1	17108337	17249642	141305	Gain	17106814-17116867	17241175-17251240	80	LCR22-2'	aCGH

2	17108337	17249642	141305	Loss	17106814-17116867	17241175-17251240	80	LCR22-2'	aCGH

3	21346546	21412512	65966	Loss	21344443-21350044	21410029-21415309	42	LCR22-5'	sequencing

4	21350920	21372688	21768	Loss	21350334-21351679	21371980-21373335	52	LCR22-5'	sequencing

5	21359730	21420692	60962	Loss	21359721-21367895	21420384-21428665	58	LCR22-5'	sequencing

6	21364900	21425105	60205	Loss	21359721-21367895	21420384-21428665	58	LCR22-5'	sequencing

7	21378929	21437071	58142	Loss	21376652-21382799	21434410-21440989	94	LCR22-5'	sequencing

8	21381357	21439879	58522	Loss	21376652-21382799	21434410-21440989	94	LCR22-5'	sequencing

9	21430381	21494380	63999	Loss	21429486-21431884	21493407-21495966	71	LCR22-5'	sequencing

10	22245900	22313362	67462	Loss	22244487-22248921	22309895-22314095	98	LCR22-6'	Paired End Mapping

11	22247409	22311770	64361	Loss	22244487-22248921	22309895-22314095	98	LCR22-6'	aCGH

12	22291744	22295976	4232	Loss	22288656-22292395	22292394-22296670	6	LCR22-6'	sequencing

13	22626028	22660855	34827	Loss	22621224-22626431	22659321-22664520	8	LCR22-7'	aCGH

### CNVs flanked by Alu elements

**Figure 6:**
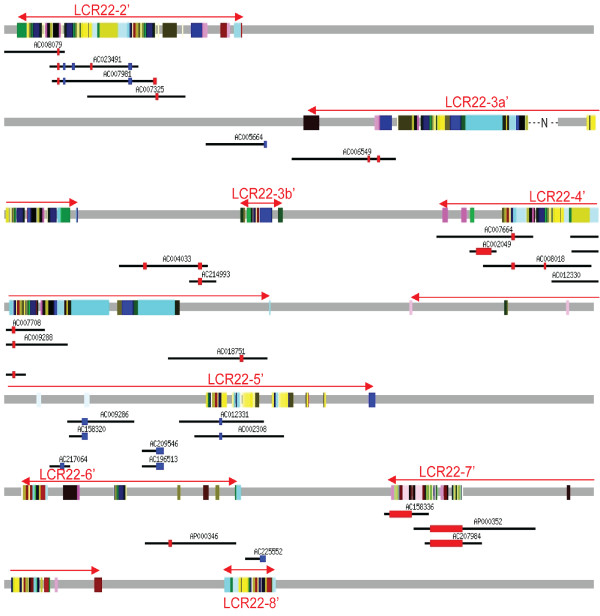
**Distribution of BAC and other genomic clones and CNVs derived from them**. A total of 191 clones were mapped to the 22q11.2 region in the human reference genome, resulting 28 CNVs (blue for gain and red for loss). Only clones with CNVs are shown here to simplify the figure. Coordinates of these CNVs are available in Table 3.

In addition to publicly available CNV data, we have employed a BAC/PAC clone mapping approach to uncover CNVs on 22q11.2. We collected DNA sequence from 191 large insert clones, cosmids, PACs and BACs, from GenBank, and their alignment to the reference 22q11.2 sequence revealed 10 insertions and 18 deletions of sizes > 200 bp (Figure 6; Table 3). Four of these CNVs were more than 10 kb in size, one 11.9 kb insertion, and the other three were 25.9 kb, 37.1 kb and 54.3 kb deletions (Table 3). A total of 11 of the remaining CNVs were relatively small (< 1 kb), whereas 13 of them were intermediate in length (1 ~10 kb). Moreover, 19 and nine of these CNVs (p < 0.001) were in the duplicated and unique regions, respectively, providing additional line of evidence that SDs show significant genetic variation.

**Table 3:** A total of 28 CNVs derived from alignment of clones to 22q11.2 (see Figure 7B, C for illustration)

ID	CNV start	CNV End	Length (bp)	Gain/Loss	IDs of the Corresponding Clones	Breakpoint Feature (one for gain, but left-right for loss)
1	17101986	17101986	269	Gain	AC007981	AT_rich

2	17101994	17101994	367	Gain	AC023491	AT_rich

3	17117298	17117298	216	Gain	AC023491	Na

4	17213332	17213332	1467	Gain	AC007981	AluY/AluSg

5	18443137	18443137	849	Gain	AC005664	AluY

6	21045602	21045602	6065	Gain	AC217064	L1PB1

7	21082588	21082588	9100	Gain	AC009286	L2

8	21208336	21208336	11915	Gain	AC209546	AluY

9	21317338	21317338	322	Gain	AC012331	AluSg

10	22384885	22384885	8981	Gain	AC225552	LTR43

11	17091525	17092039	514	Loss	AC008079/AC007981/AC023491	L1M4c-L1M4c

12	17147850	17148359	509	Loss	AC023491	AluY-AluSx

13	17255132	17256943	1811	Loss	AC007981	AluSx-Na

14	17259091	17259821	730	Loss	AC007325	(TATAA)n-(TA)n

15	18619242	18619474	232	Loss	AC006549	(CACCAT)n-Na

16	18635423	18636050	627	Loss	AC006549	Na-Na

17	19188424	19189864	1440	Loss	AC004033	Na-AluSc

18	19280623	19283840	3217	Loss	AC214993	MLT2B1-AluJb

19	19751004	19776895	25891	Loss	AC002049	AluY-AluJb

20	19813055	19814522	1467	Loss	AC008018	AluY-AluY

21	19867770	19868094	324	Loss	AC008018	Na-Na

22	19964433	19965896	1463	Loss	AC007708	AluY-AluY

23	19964603	19966056	1453	Loss	AC009288	AluY-AluY

24	19964603	19966061	1458	Loss	AC012330	AluY-AluY

25	20352325	20353913	1588	Loss	AC018751	AluSx-AluSx

26	22230772	22233234	2462	Loss	AP000346	AluJb-Na

27	22604144	22641299	37155	Loss	AC158336	Na-Na

28	22673453	22727704	54251	Loss	AP000352	Subunit/GSTTP (22673143-22673594, 22727394-22727845)

**Figure 7:**
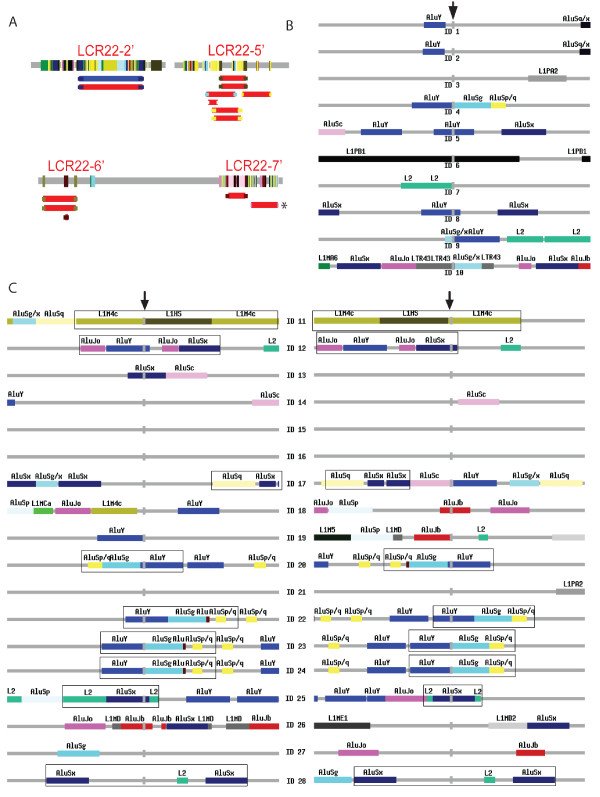
**Many CNVs are flanked by paralogous subunits and/or Alu SINES**. (A) A total of 13 previously detected CNVs have their endpoints located to paralogous subunits. All subunits are colored as Figure 2, in addition, with blue color for gain CNVs and red for loss CNVs. One CNV marked with a "*" is found by our clone mapping (Figure 6). Sequence features around (± 1 kb) the insertion sites of ten gain CNVs (B) or the two breakpoints of 18 loss CNVs (C) from current clone mapping analysis. In (B) and (C) arrows point to the breakpoints and coordinates and other detailed information is in Table 3.

As the CNVs from large insert clone mapping (Figure 6) identified breakpoints down to the base-pair level, we further characterized the sequence in the vicinity of them. We found that 15 (54%) of the 28 CNVs had at least one breakpoint terminating at an *Alu *repeat (Table 3; Figure 7). Again, the most prevalent group was *AluY*, which was next to nine CNVs. The enrichment of *AluY *is highly significant (p < 0.001) based on simulation. Furthermore, we have compared the sequence similarity between the two short sequences immediately adjacent (±10 bp) to the two breakpoints of each CNV and found that nine (50%) of the 18 deletion CNVs might be the result of meiotic NAHR events (Table 3). A pair of *AluY *repeats (*AluY-AluY*) was located at the two breakpoints of four of the nine CNVs (Figure 7). Likewise, same repeats, one in the form of *AluSx-AluSx *and the other in the form of *L1-L1 LINE*, were next to the breakpoints of two CNVs. Another two CNVs may be results of NAHR mediated by two different sub-groups of *Alu*, one with *AluY-AluSx *and the other with *AluY-AluJo*. More interestingly, we found that the generation of one CNV (chr22:22,673,453-22,727,704) may have been mediated by paralogous subunits. These subunits are part of two non-processed pseudogenes derived from glutathione S-transferase theta (*GSTT*) gene. In summary, our analysis suggests that *AluY *plays a key role in the genome dynamics of 22q11.2 by mediating the generations of both SDs and CNVs, largely through NAHR.

## Discussion

Segmental duplications on 22q11.2 are some of the most complicated SDs in the human genome but the value in understanding their structure and variation among humans is because they form substrates for NAHR events that can lead to gene dosage imbalance and genomic disorders. Such major disorders include VCFS/DGS, the reciprocal duplication in the same interval, and cat-eye syndrome, a partial tetrasomy [5-12]. Patients with VCFS/DGS and separately, the duplication syndrome have been ascertained for physical malformations including craniofacial and cardiac defects, but also due to cognitive or neurobehavioral disorders such as learning disabilities or schizophrenia (reviewed in: [33,34]).

To understand structure features in 22q11.2 and mechanisms by which SDs on 22q11.2 confer genetic variation and CNV formation, we have decomposed them into fundamental duplication subunits to investigate sequence shuffling within LCR22s, past duplication events, and current duplications (i.e., CNVs). Our key finding is that some subunits are highly active in duplications and *AluY*, a young repeat emerging very recently during primate evolution [15], is significantly associated with them, suggesting subunits next to *AluY *or *AluY *itself may be responsible for historic and current genomic rearrangements in SDs on 22q11.2. We also found that LCR22-2', LCR22-3a', and LCR22-4', the three young blocks implicated most frequently in genomic disorders, contained duplicated sequences emerging more recently than the other SDs on 22q11.2. The high sequence identity among these three blocks may explain why most pathogenic deletions are mapped to these three blocks. Alternatively, phenotypes arising from genes whose function is sensitive to altered copy number will incur an ascertainment bias of these deletions.

Overall, SDs on 22q11.2 share many similar and known features with other SDs in the human genome in addition to *Alu *enrichment discussed above. The cluster of SDs in eight blocks is a testament of "preferential attachment" [35] or "duplication shadowing", meaning that unique regions next to SDs are ~10 times more likely to be duplicated than random regions [16]. Our syntenic analysis also suggests that most (~70%) 22q11.2 SDs are shared between human and chimpanzee but only a small proportion of them is shared between human and macaque, a finding that is entirely consistent with previous proposition that SD activity increases after the divergence of African great apes (chimpanzee, gorilla and human) from the Asian great ape (orangutan) [29,36]. Our current study used SDs in the human chromosome 22q11.2 as reference, and thus sequence amplifications occurring specifically in the chimpanzee, gorilla and macaque genomes were not analyzed, but previous studies have observed amplifications specific to those lineages [24,29].

We found that *Alu *elements, especially young *AluY*, were enriched in the immediate adjacent regions of frequently duplicated sequences (subunits, duplication loci, and CNVs). Our results thus extend previous findings that have shown the presence of *Alu *elements at the endpoints of SDs at a higher frequency than expected by chance (24% vs 10%) [15,16] and more specifically a three-fold enrichment of *Alu *in the junctions of LCR16 [37]. In addition, we have previously shown that both simple and complex *Alu*-mediated duplications stimulated by crossovers at the ends of *Alu *elements may have contributed to the formation of unprocessed pseudogenes from the four LCR22 genes [14,26]. In Figure 7, we show that most of the breakpoints are at the ends, while a subset in the middle of *Alu *elements, suggesting that homology based alignment is essential for CNV and SD formation, but likely there two distinct molecular mechanisms responsible, L1 endonuclease-mediated retrotransposition and NAHR events [14].

Taken together, these results suggest that *de novo *and disease-implicated recombination events between LCR22-2 and LCR22-4 may not occur randomly but more frequently at *Alu*-embedding subsequences, an interesting hypothesis deserving of further investigation in the future. In this regard, it is important to mention that common breakpoints found in the rearrangement of distal LCR22 blocks in two patients [38] is located to one of the highly active subunits identified in this study (the subunit in red color at the end of the first duplication group in Figure 2B), supporting that our map of duplications could be useful for studying human genomic disorders. Some interesting directions to further explore our findings are, (i) *Alu *sequences may be preferential sites of double strand breakage after homology based alignment [14], and (ii) chromatin modification in the vicinity of *Alu *sequences may make a region prone for duplications as local chromatin structure (e.g., accessibility) is an important factor influencing DNA duplication and its subsequent evolution [19,39]. Along the same line, we should note here that *AluY *insertion sites have been reported to show elevated recombination rates [40]. Furthermore, we found that the recently discovered recombination hotspot motif (CCNCCNTNNCCNC) [41] was significantly enriched at the breakpoints of both SD subunits (1.6-fold enrichment, p= 0.026) and CNVs (2.3-fold enrichment, p = 0.016; using data derived from our BAC mapping analysis) in 22q11.2.

In this report we describe our efforts of interpreting large duplication events and employing cross-genome comparison to narrow down the potential evolutionary periods. Our work provides some important insights, but also highlights the complexity and challenge ahead. It is difficult to identify and time individual duplication events that have left the mosaic genome architecture of LCR22s as shown in Figure 2 and 3. During our study, we explored other complementary approaches but without significant success. For example, we examined phylogenetic relationship of orthologous and paralogous subunits using both human and syntenic sequences from other primates, but the resulting phylogenetic trees were often difficult to interpret or were only able to help resolve the precise emerging times of a limited number of duplication subunits, suggesting extensive gene conversion may have occurred among paralogous subunits. It is clear that, to achieve more from cross-species comparison, we would need more great Ape genomes to be sampled more densely at finer scale of evolutionary time. Otherwise, it is like trying to re-construct primate evolution with too many missing fossils. Additionally, we believe that low-coverage (e.g., 1-4x) sequencing will provide limited help. As an example, the syntenic regions of human LCR22-2' and LCR22-4' in the reference chimpanzee genome contain some large gaps so that we were unable to extract critical evolutionary information for certain important duplication events in these two blocks - their synteny was considered ambiguous in our analysis. Our study suggests that special care must be taken for comparing duplicated regions across genomes to resolve the ambiguity between overlapping alleles and duplicated paralogs with high level of sequence identity (>98%). In order to employ comparative genomics to study the molecular mechanism of genome disorders involving in complex duplicated regions, we propose that an alternative and probably more effective strategy is to establish a good reference of common CNVs for those regions. In the case of LCR22s, this will mean specifically targeting LCR22s for deep sequencing with a large number of human samples. One critical challenge is how to distinguish duplications (i.e., CNVs) with high level of sequence identity (>98%) from allelic overlapping when sequence reads are too short to be aligned uniquely or assembled correctly.

## Conclusions

Our detailed analysis of the human 22q11.1 region showed that many of its duplicated sequences emerged recently through both small and large-scale duplications. We also found a great number of copy number variations in 22q11.2 and some of them may be generated by DNA recombination mediated by paralogous subunits or young SINE, *AluY*. Our results suggest that genomic rearrangements in 22q11.2 do not occur randomly and active duplicated subunits, subunits adjacent to *Alus*, and *AluY *elements all play a role in making some sequences better substrates for recombination.

## Methods

### Segmental duplications, subunit identification and classification

A total of 202 pairs of segmental duplications from human chr22:17,000, 000-24, 000,000 (hg18) were obtained from the segmental duplication track in the UCSC genome browser (http://genome.ucsc.edu). SDs involving sequences outside of 22q11.2 were not included, as our goal was to study the intra-LCR22 duplications implicated in human disorders. The pair-wise alignment information of these SDs was provided to the program RepeatGluer [22] for decomposing the 404 SDs into 523 non-overlapping duplication subunits. This approach is motivated by previous study [21,23]; a comparison of our subunits with those defined previously by Jiang *et al *[23] with the same algorithm found that 69 subunits were only defined by us although the breakpoints for 84% of the common subunits differed <200 bp in the two definition (see Additional file 4, Figure S3 for details). This data suggest that inclusion of SDs outside 22q11.2 could have some impacts on our results.

Individual subunit sequences were classified to 122 families based on their sequence homology (>90% identity) (Figure 1) and segregated to eight duplication blocks based on their physical distance. In the latter analysis, we took the previous definition of eight LCR22 blocks as a guideline and assigned adjacent subunits (<500 kb) to the same block. The selection of 500 kb was to include as many SDs in the eight blocks as possible and to use one consistent threshold for all blocks. As a result, some non-SD sequences embedded in SDs were included in LCR22-5', -6', and 7'.

### Constructing hierarchical map of putative duplication events

As shown in Figure 1, SD pair-wise alignment data are a good summary of the paralogous relationship for a pair or a group of SDs but they do not directly reveal the underlying historical duplication events. Our overall strategy for re-constructing past intra-LCR22 duplication events was to first merge overlapping duplicons to form individual duplication locus, and then group duplication loci based on SD pair-wise relationship, and then project all duplication loci to the largest duplication locus on a group based on sequence alignment (Figure 1C). The resulting alignment map illustrated the hierarchical order of putative duplications (Figure 2), as the donor and acceptor of a duplication event would have the same subunits arranged in the identical order, unless disruption had occurred. With the caveat that sometimes duplications could produce a merged sequence and uncertainty of donor assignment grew for shorter SDs, we have only interpreted this map for duplications involving long sequence and multiple subunits, i.e., at the top of the hierarchy.

### Syntenic analysis of SDs on 22q11.2

Multiple genome alignment data from the Ensembl site (http://www.ensembl.org/info/docs/api/compara/index.html) was used to search syntenic sequences for human 22q11.2 SD sequences. More specifically, the pair-wise alignment data was generated by the program Blastz-net [42] between human and each of other three primate species: chimp, orangutan and macaque. We first analyzed the synteny of unique sequences in human 22q11.2 and utilized the result to establish a global syntenic reference map. Then, the occurrence of aligned sequence in the expected syntenic location on this map was considered as evidence that a human duplication subunit (or entire SD) had a syntenic partner in a subject species. To account for the confounding factors in sequencing and assembling duplicated sequences, we also considered syntenic sequence present if the syntenic location of a human sequence was a stretch of "N" nucleotides and homologous sequence was located in unassembled contigs (e.g., chr_random). Furthermore, we utilized the syntenic locations of two unique sequences (i.e., landmarks) bracketing each human duplicated sequence to help identify missing syntenic relationship; the synteny of a human sequence was considered absent if no aligned sequence was found in the expected syntenic location and the distance of the two adjacent landmarks in the target genome was 2x shorter than that in the human 22q11.2. All these measurements certainly cannot account for the draft nature of the non-human genomes fully, so some degree of uncertainty is expected from our syntenic analysis. On the other hand, we found supportive evidence for our syntenic results from all 15 chimp BACs available in GenBank and mapped onto LCR22. Specifically, 9 of these 15 chimp BACs were in the regions where 22 subunits were found missing in chimp; all of these 22 subunits were confirmed to be absent from their respective BAC sequences.

We also carried out similar syntenic analysis using either in-house constructed global alignments with BLASTZ [42] or cross-species liftOver data from the UCSC browser and obtained similar result, and thus was not discussed here. The comparative data of segmental duplications in human, chimp, orangutan and macaque based on Whole Genome Shotgun Sequences Detection was obtained from a previous study [29].

### Analysis of copy number variations in LCR22s

Previously annotated CNVs were collected from three sources (the Database of Genomic Variants (http://projects.tcag.ca/variation/, downloaded on August 2009); [31,43]). Also, 191 BACs or PACs were downloaded from the NCBI GenBank and aligned to the human reference genome using the FASTA software package [44]. Gaps of > 200 bp in the alignments were defined as CNVs and annotated as insertions or deletions with respect to the reference genome.

### Analysis of repetitive elements

In the search of sequence features associated with either subunits or CNVs, we annotated the ± 10 bp sequences adjacent to breakpoints as suggested previously [15,45]. Our annotation included micro-sequence homology detection and the presence of repetitive elements as defined by RepeatMasker. Here, micro-homology was defined as > 80% identity of 10-bp sequences. To assess the statistical significance of the *Alu *enrichment in subunits (or CNVs), we randomly put these subunits in the human genome and calculated the expected number of subunits with *Alu *elements. After repeating this procedure 1,000 times, we derived an empirical p-value for *Alu *enrichment. This method was also employed to assess the significance of gene and pseudogene enrichment in SD regions.

## Abbreviations

LCR: low copy repeats; SD: segmental duplication; CNV: copy number variation.

## Authors' contributions

BM and DZ conceived of the study. XG and DZ designed the computational experiments and XG performed the analysis. LF and BM carried out the PCR assays. XG, DZ and BM interpreted the data and wrote the manuscript. All authors read and approved the final manuscript.

## Supplementary Material

Additional file 1 **Supplementary Figure S1**. The divergence of segmental duplications (SDs) in 22q11.2 is negatively correlated with their sequence length. Click here for file

Additional file 2 **Supplementary Table S1**. A list of all 523 duplication subunits resulting from our decomposition of SDs in 22q11.2, with their paralogous relationship identified by family indexes. Click here for file

Additional file 3 **Supplementary Figure S2**. PCR Analysis Confirmed a Duplication Event Absent in the Macaque Genome. We carried out PCR analysis for a duplicated sequence (chr22:21,293,079-21,327,588; hg18) that was predicted to be specific to the human and chimp genomes from our sytenic analysis. Click here for file

Additional file 4 **Supplementary Figure S3**. Comparison of Currently Defined SD Subunits with those Defined by Jiang et al. in Previous Work [ref [21] and ref [23]]. Click here for file

## References

[B1] Bailey JA Gu Z Clark RA Reinert K Samonte RV Schwartz S Adams MD Myers EW Li PW Eichler EE Recent segmental duplications in the human genome Science 2002 297 5583 1003 1007 10.1126/science.1072047 12169732

[B2] She X Jiang Z Clark RA Liu G Cheng Z Tuzun E Church DM Sutton G Halpern AL Eichler EE Shotgun sequence assembly and recent segmental duplications within the human genome Nature 2004 431 7011 927 930 10.1038/nature03062 15496912

[B3] Bailey JA Yavor AM Massa HF Trask BJ Eichler EE Segmental duplications: organization and impact within the current human genome project assembly Genome Res 2001 11 6 1005 1017 10.1101/gr.GR-1871R 11381028 PMC311093

[B4] Halford S Wadey R Roberts C Daw SC Whiting JA O'Donnell H Dunham I Bentley D Lindsay E Baldini A Isolation of a putative transcriptional regulator from the region of 22q11 deleted in DiGeorge syndrome, Shprintzen syndrome and familial congenital heart disease Hum Mol Genet 1993 2 12 2099 2107 10.1093/hmg/2.12.2099 8111380

[B5] Edelmann L Pandita RK Morrow BE Low-copy repeats mediate the common 3-Mb deletion in patients with velo-cardio-facial syndrome Am J Hum Genet 1999 64 4 1076 1086 10.1086/302343 10090893 PMC1377832

[B6] McDermid HE Morrow BE Genomic disorders on 22q11 Am J Hum Genet 2002 70 5 1077 1088 10.1086/340363 11925570 PMC447586

[B7] Shprintzen RJ Goldberg RB Lewin ML Sidoti EJ Berkman MD Argamaso RV Young D A new syndrome involving cleft palate, cardiac anomalies, typical facies, and learning disabilities: velo-cardio-facial syndrome Cleft Palate J 1978 15 1 56 62 272242

[B8] DiGeorge AM Harley RD The association of aniridia, Wilms's tumor, and genital abnormalities Trans Am Ophthalmol Soc 1965 63 64 69 4285715 PMC1310184

[B9] Hassed SJ Hopcus-Niccum D Zhang L Li S Mulvihill JJ A new genomic duplication syndrome complementary to the velocardiofacial (22q11 deletion) syndrome Clin Genet 2004 65 5 400 404 10.1111/j.0009-9163.2004.0212.x 15099348

[B10] Ensenauer RE Adeyinka A Flynn HC Michels VV Lindor NM Dawson DB Thorland EC Lorentz CP Goldstein JL McDonald MT Microduplication 22q11.2, an emerging syndrome: clinical, cytogenetic, and molecular analysis of thirteen patients Am J Hum Genet 2003 73 5 1027 1040 10.1086/378818 14526392 PMC1180483

[B11] Zackai EH Emanuel BS Site-specific reciprocal translocation, t(11;22) (q23;q11), in several unrelated families with 3:1 meiotic disjunction Am J Med Genet 1980 7 4 507 521 10.1002/ajmg.1320070412 7211960

[B12] Knoll JH Asamoah A Pletcher BA Wagstaff J Interstitial duplication of proximal 22q: phenotypic overlap with cat eye syndrome Am J Med Genet 1995 55 2 221 224 10.1002/ajmg.1320550214 7717422

[B13] Shaikh TH Kurahashi H Saitta SC O'Hare AM Hu P Roe BA Driscoll DA McDonald-McGinn DM Zackai EH Budarf ML Chromosome 22-specific low copy repeats and the 22q11.2 deletion syndrome: genomic organization and deletion endpoint analysis Hum Mol Genet 2000 9 4 489 501 10.1093/hmg/9.4.489 10699172

[B14] Babcock M Pavlicek A Spiteri E Kashork CD Ioshikhes I Shaffer LG Jurka J Morrow BE Shuffling of genes within low-copy repeats on 22q11 (LCR22) by Alu-mediated recombination events during evolution Genome Res 2003 13 12 2519 2532 10.1101/gr.1549503 14656960 PMC403794

[B15] Bailey JA Liu G Eichler EE An Alu transposition model for the origin and expansion of human segmental duplications Am J Hum Genet 2003 73 4 823 834 10.1086/378594 14505274 PMC1180605

[B16] Bailey JA Eichler EE Primate segmental duplications: crucibles of evolution, diversity and disease Nat Rev Genet 2006 7 7 552 564 10.1038/nrg1895 16770338

[B17] Ohno S Evolution by gene duplication 1970 London: George Allen and Unwin

[B18] Lynch M Conery JS The evolutionary fate and consequences of duplicate genes Science 2000 290 5494 1151 1155 10.1126/science.290.5494.1151 11073452

[B19] Zheng D Asymmetric histone modifications between the original and derived loci of human segmental duplications Genome Biol 2008 9 7 R105 10.1186/gb-2008-9-7-r105 18598352 PMC2530858

[B20] Harrow J Denoeud F Frankish A Reymond A Chen CK Chrast J Lagarde J Gilbert JG Storey R Swarbreck D GENCODE: producing a reference annotation for ENCODE Genome Biol 2006 7 Suppl 1 S4 1 9 10.1186/gb-2006-7-s1-s4 PMC1810553 16925838

[B21] Jiang Z Tang H Ventura M Cardone MF Marques-Bonet T She X Pevzner PA Eichler EE Ancestral reconstruction of segmental duplications reveals punctuated cores of human genome evolution Nat Genet 2007 39 11 1361 1368 10.1038/ng.2007.9 17922013

[B22] Pevzner PA Tang H Tesler G De novo repeat classification and fragment assembly Genome Res 2004 14 9 1786 1796 10.1101/gr.2395204 15342561 PMC515325

[B23] Jiang Z Hubley R Smit A Eichler EE DupMasker: a tool for annotating primate segmental duplications Genome Res 2008 18 8 1362 1368 10.1101/gr.078477.108 18502942 PMC2493431

[B24] Babcock M Yatsenko S Hopkins J Brenton M Cao Q de Jong P Stankiewicz P Lupski JR Sikela JM Morrow BE Hominoid lineage specific amplification of low-copy repeats on 22q11.2 (LCR22s) associated with velo-cardio-facial/digeorge syndrome Hum Mol Genet 2007 16 21 2560 2571 10.1093/hmg/ddm197 17675367

[B25] Edelmann L Pandita RK Spiteri E Funke B Goldberg R Palanisamy N Chaganti RS Magenis E Shprintzen RJ Morrow BE A common molecular basis for rearrangement disorders on chromosome 22q11 Hum Mol Genet 1999 8 7 1157 1167 10.1093/hmg/8.7.1157 10369860

[B26] Pavlicek A House R Gentles AJ Jurka J Morrow BE Traffic of genetic information between segmental duplications flanking the typical 22q11.2 deletion in velo-cardio-facial syndrome/DiGeorge syndrome Genome Res 2005 15 11 1487 1495 10.1101/gr.4281205 16251458 PMC1310636

[B27] Bailey JA Yavor AM Viggiano L Misceo D Horvath JE Archidiacono N Schwartz S Rocchi M Eichler EE Human-specific duplication and mosaic transcripts: the recent paralogous structure of chromosome 22 Am J Hum Genet 2002 70 1 83 100 10.1086/338458 11731936 PMC419985

[B28] Hubbard TJ Aken BL Ayling S Ballester B Beal K Bragin E Brent S Chen Y Clapham P Clarke L Ensembl 2009 Nucleic Acids Res 2009 37 Database D690 697 10.1093/nar/gkn828 19033362 PMC2686571

[B29] Marques-Bonet T Kidd JM Ventura M Graves TA Cheng Z Hillier LW Jiang Z Baker C Malfavon-Borja R Fulton LA A burst of segmental duplications in the genome of the African great ape ancestor Nature 2009 457 7231 877 881 10.1038/nature07744 19212409 PMC2751663

[B30] Bennett EA Keller H Mills RE Schmidt S Moran JV Weichenrieder O Devine SE Active Alu retrotransposons in the human genome Genome Res 2008 18 12 1875 1883 10.1101/gr.081737.108 18836035 PMC2593586

[B31] Conrad DF Pinto D Redon R Feuk L Gokcumen O Zhang Y Aerts J Andrews TD Barnes C Campbell P Origins and functional impact of copy number variation in the human genome Nature 2009 464 7289 704 712 10.1038/nature08516 19812545 PMC3330748

[B32] Iafrate AJ Feuk L Rivera MN Listewnik ML Donahoe PK Qi Y Scherer SW Lee C Detection of large-scale variation in the human genome Nat Genet 2004 36 9 949 951 10.1038/ng1416 15286789

[B33] Bassett AS Scherer SW Brzustowicz LM Copy Number Variations in Schizophrenia: Critical Review and New Perspectives on Concepts of Genetics and Disease Am J Psychiatry 2010 167 8 899 914 10.1176/appi.ajp.2009.09071016 20439386 PMC3295834

[B34] Karayiorgou M Simon TJ Gogos JA 22q11.2 microdeletions: linking DNA structural variation to brain dysfunction and schizophrenia Nat Rev Neurosci 2010 11 6 402 416 10.1038/nrn2841 20485365 PMC2977984

[B35] Kim PM Lam HY Urban AE Korbel JO Affourtit J Grubert F Chen X Weissman S Snyder M Gerstein MB Analysis of copy number variants and segmental duplications in the human genome: Evidence for a change in the process of formation in recent evolutionary history Genome Res 2008 18 12 1865 1874 10.1101/gr.081422.108 18842824 PMC2593581

[B36] Marques-Bonet T Girirajan S Eichler EE The origins and impact of primate segmental duplications Trends Genet 2009 25 10 443 454 10.1016/j.tig.2009.08.002 19796838 PMC2847396

[B37] Johnson ME Cheng Z Morrison VA Scherer S Ventura M Gibbs RA Green ED Eichler EE Recurrent duplication-driven transposition of DNA during hominoid evolution Proc Natl Acad Sci USA 2006 103 47 17626 17631 10.1073/pnas.0605426103 17101969 PMC1693797

[B38] Shaikh TH O'Connor RJ Pierpont ME McGrath J Hacker AM Nimmakayalu M Geiger E Emanuel BS Saitta SC Low copy repeats mediate distal chromosome 22q11.2 deletions: sequence analysis predicts breakpoint mechanisms Genome Res 2007 17 4 482 491 10.1101/gr.5986507 17351135 PMC1832095

[B39] Zheng D Gene duplication in the epigenomic era: Roles of chromatin modificationsepigenetics 2008 3 5 250 253 10.4161/epi.3.5.6991 19013828

[B40] Witherspoon DJ Watkins WS Zhang Y Xing J Tolpinrud WL Hedges DJ Batzer MA Jorde LB Alu repeats increase local recombination rates BMC Genomics 2009 10 530 10.1186/1471-2164-10-530 19917129 PMC2785838

[B41] Myers S Freeman C Auton A Donnelly P McVean G A common sequence motif associated with recombination hot spots and genome instability in humans Nat Genet 2008 40 9 1124 1129 10.1038/ng.213 19165926

[B42] Schwartz S Kent WJ Smit A Zhang Z Baertsch R Hardison RC Haussler D Miller W Human-mouse alignments with BLASTZ Genome Res 2003 13 1 103 107 10.1101/gr.809403 12529312 PMC430961

[B43] Kidd JM Cooper GM Donahue WF Hayden HS Sampas N Graves T Hansen N Teague B Alkan C Antonacci F Mapping and sequencing of structural variation from eight human genomes Nature 2008 453 7191 56 64 10.1038/nature06862 18451855 PMC2424287

[B44] Pearson WR Flexible sequence similarity searching with the FASTA3 program package Methods Mol Biol 2000 132 185 219 10547837 10.1385/1-59259-192-2:185

[B45] Korbel JO Urban AE Affourtit JP Godwin B Grubert F Simons JF Kim PM Palejev D Carriero NJ Du L Paired-end mapping reveals extensive structural variation in the human genome Science 2007 318 5849 420 426 10.1126/science.1149504 17901297 PMC2674581

