# Supplementary material for: Characterization of the past and current duplication activities in the human 22q11.2 region
Source: BMC Genomics. 2011 Jan 26;12:71. doi: 10.1186/1471-2164-12-71 (PMC3040729; doi:10.1186/1471-2164-12-71)

Additional File 1.

Supplementary Figure S1. The divergence of segmental duplications (SDs) in 22q11.2 is negatively correlated with their sequence length. A, The histogram of lengths of SD duplicons. B, The histogram of divergences of SD pairs. C, The anti-correlation between sequence divergence and alignment length of SDs.

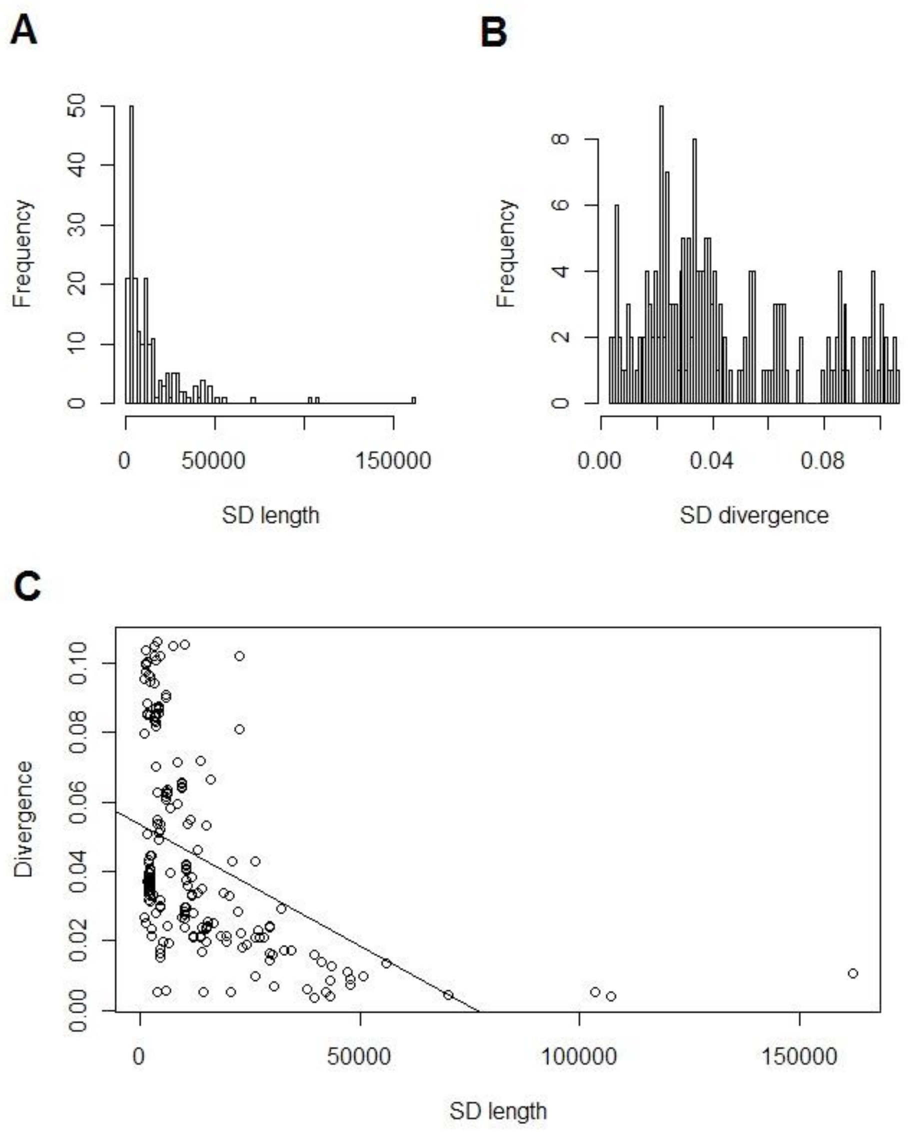

Supplement: Additional file 1 — Supplementary Figure S1. The divergence of segmental duplications (SDs) in 22q11.2 is negatively correlated with their sequence length. [file 1471-2164-12-71-S1.PDF]
