# Supplementary material for: Characterization of the past and current duplication activities in the human 22q11.2 region
Source: BMC Genomics. 2011 Jan 26;12:71. doi: 10.1186/1471-2164-12-71 (PMC3040729; doi:10.1186/1471-2164-12-71)
Supplement: Additional file 2 — Supplementary Table S1. A list of all 523 duplication subunits resulting from our decomposition of SDs in 22q11.2, with their paralogous relationship identified by family indexes. [file 1471-2164-12-71-S2.PDF]

Additional File 2.

Supplementary Table S1. A list of all 523 duplication subunits resulting from our decomposition of SDs in 22q11.2, with their paralogous relationship identified by family indexes.

Family Index	Start	End	Block Assignment
1	21367894	21368186	LCR22-5'
1	21428664	21429487	LCR22-5'
1	21462285	21463134	LCR22-5'
1	21492572	21493408	LCR22-5'
2	18508559	18534340	LCR22-3a'
2	22050745	22074529	LCR22-6'
3	18853205	18857535	LCR22-3a'
3	20051910	20056240	LCR22-4'
3	22905288	22910915	LCR22-7'
4	17129118	17129389	LCR22-2'
4	17216351	17216610	LCR22-2'
4	18734162	18734434	LCR22-3a'
4	18958146	18958415	LCR22-3a'
4	19376226	19376490	LCR22-3b'
4	19809762	19810020	LCR22-4'
4	19880149	19880422	LCR22-4'
4	19969078	19969334	LCR22-4'
4	21314398	21314656	LCR22-5'
4	22979887	22980145	LCR22-7'
4	23382575	23382834	LCR22-8'
5	17097050	17097453	LCR22-2'
5	19849825	19850208	LCR22-4'
6	22288656	22292395	LCR22-6'
6	22292394	22296670	LCR22-6'
7	22639774	22641745	LCR22-7'
7	22643970	22645947	LCR22-7'
8	22621224	22626431	LCR22-7'
8	22659321	22664520	LCR22-7'
8	22696216	22701085	LCR22-7'
9	21993881	21997289	LCR22-6'
9	22994779	22999782	LCR22-7'
10	17367203	17377993	LCR22-2'
10	23103562	23115236	LCR22-7'
11	17218497	17223406	LCR22-2'

11	19378427	19383334	LCR22-3b'
11	19802967	19807876	LCR22-4'
11	19971220	19976129	LCR22-4'
11	21307547	21312460	LCR22-5'
11	21986635	21991523	LCR22-6'
11	22982074	22986988	LCR22-7'
11	23375726	23380639	LCR22-8'
12	22011886	22020337	LCR22-6'
12	23018262	23026664	LCR22-7'
12	23395902	23404383	LCR22-8'
13	21997538	21998101	LCR22-6'
13	23000033	23000575	LCR22-7'
14	21371089	21371981	LCR22-5'
14	21407760	21408661	LCR22-5'
14	21431883	21432780	LCR22-5'
14	21465675	21466893	LCR22-5'
15	17401981	17403012	LCR22-2'
15	18868779	18869810	LCR22-3a'
15	18989599	18990630	LCR22-3a'
15	20039630	20040661	LCR22-4'
16	22619582	22619853	LCR22-7'
16	22665885	22666156	LCR22-7'
16	22712165	22712454	LCR22-7'
16	22718132	22718421	LCR22-7'
17	17059237	17060379	LCR22-2'
17	17105673	17106815	LCR22-2'
17	17251239	17252381	LCR22-2'
17	18710648	18711789	LCR22-3a'
17	18981070	18982211	LCR22-3a'
17	19027407	19028549	LCR22-3a'
17	19856365	19857507	LCR22-4'
17	20003874	20005016	LCR22-4'
18	22674049	22674181	LCR22-7'
18	22682662	22682810	LCR22-7'
19	21295717	21296105	LCR22-5'
19	21296701	21297747	LCR22-5'
19	22964515	22965031	LCR22-7'
19	22965150	22965330	LCR22-7'
19	23365125	23365496	LCR22-8'
19	23365792	23366809	LCR22-8'
20	17335064	17337911	LCR22-2'

20	18635849	18638727	LCR22-3a'
20	22074948	22077444	LCR22-6'
21	17070157	17073199	LCR22-2'
21	17092538	17095586	LCR22-2'
21	18700738	18703776	LCR22-3a'
21	19014510	19017552	LCR22-3a'
21	19845449	19848497	LCR22-4'
21	20012716	20015753	LCR22-4'
22	19694656	19702643	LCR22-4'
22	19726341	19734780	LCR22-4'
23	22003646	22003974	LCR22-6'
23	23005341	23005650	LCR22-7'
23	23387622	23387943	LCR22-8'
24	22614473	22614922	LCR22-7'
24	22619072	22619355	LCR22-7'
24	22666898	22667183	LCR22-7'
24	22671614	22672061	LCR22-7'
24	22676962	22677407	LCR22-7'
24	22713670	22714136	LCR22-7'
24	22719581	22720026	LCR22-7'
24	22725548	22725993	LCR22-7'
24	22612126	22612578	LCR22-7'
24	22616814	22617281	LCR22-7'
24	22668458	22668922	LCR22-7'
24	22673143	22673594	LCR22-7'
24	22680682	22681133	LCR22-7'
24	22715446	22715911	LCR22-7'
24	22721427	22721878	LCR22-7'
24	22727394	22727845	LCR22-7'
25	22674180	22674593	LCR22-7'
25	22682810	22683275	LCR22-7'
25	22616144	22616553	LCR22-7'
25	22620761	22621162	LCR22-7'
25	22664582	22664983	LCR22-7'
25	22669185	22669589	LCR22-7'
25	22710530	22710941	LCR22-7'
25	22716497	22716904	LCR22-7'
25	22722464	22722873	LCR22-7'
26	22607356	22612127	LCR22-7'
26	22691850	22694959	LCR22-7'
27	17337910	17344243	LCR22-2'

27	18629558	18635850	LCR22-3a'
28	17378145	17382722	LCR22-2'
28	23036513	23040755	LCR22-7'
29	21300077	21300354	LCR22-5'
29	23369134	23369411	LCR22-8'
30	20638872	20642870	LCR22-5'
30	20904965	20908964	LCR22-5'
31	22675203	22675464	LCR22-7'
31	22683964	22684258	LCR22-7'
31	22615285	22615545	LCR22-7'
31	22670188	22670448	LCR22-7'
31	22717823	22718083	LCR22-7'
31	22723790	22724050	LCR22-7'
32	22626430	22637777	LCR22-7'
32	22647945	22659322	LCR22-7'
33	19371871	19372905	LCR22-3b'
33	19813337	19814373	LCR22-4'
33	19964735	19965770	LCR22-4'
33	23386153	23387186	LCR22-8'
34	21300353	21303852	LCR22-5'
34	21979119	21982932	LCR22-6'
34	23369410	23372027	LCR22-8'
35	22011134	22011432	LCR22-6'
35	23014579	23014978	LCR22-7'
35	23395145	23395440	LCR22-8'
36	21339907	21341261	LCR22-5'
36	21358325	21359722	LCR22-5'
36	21419042	21420385	LCR22-5'
37	19041532	19043302	LCR22-3a'
37	19043376	19045249	LCR22-3a'
38	17041560	17042331	LCR22-2'
38	17123884	17124651	LCR22-2'
38	17233322	17234089	LCR22-2'
38	18728927	18729694	LCR22-3a'
38	18962887	18963654	LCR22-3a'
38	19072721	19073746	LCR22-3a'
38	19874917	19875684	LCR22-4'
38	19986043	19986810	LCR22-4'
38	22136958	22137713	LCR22-6'
39	22618097	22618267	LCR22-7'
39	22667479	22667656	LCR22-7'

39	22714736	22714942	LCR22-7'
39	22720707	22720897	LCR22-7'
40	17208543	17213033	LCR22-2'
40	19814804	19819309	LCR22-4'
40	19959817	19964304	LCR22-4'
40	21998388	22002885	LCR22-6'
40	23000572	23005044	LCR22-7'
41	22343103	22351310	LCR22-6'
41	23324444	23334132	LCR22-8'
42	21344443	21350044	LCR22-5'
42	21410029	21415309	LCR22-5'
43	19383333	19384608	LCR22-3b'
43	20400118	20401394	LCR22-4'
44	17390768	17401982	LCR22-2'
44	18857534	18868780	LCR22-3a'
44	20040660	20051911	LCR22-4'
45	22674746	22675204	LCR22-7'
45	22683448	22683964	LCR22-7'
45	22615544	22616000	LCR22-7'
45	22619853	22620308	LCR22-7'
45	22665430	22665885	LCR22-7'
45	22669733	22670189	LCR22-7'
45	22711400	22711857	LCR22-7'
45	22717367	22717824	LCR22-7'
45	22723333	22723791	LCR22-7'
46	22020336	22023902	LCR22-6'
46	23405456	23410023	LCR22-8'
47	17192498	17208544	LCR22-2'
47	19819308	19835378	LCR22-4'
47	19943748	19959818	LCR22-4'
48	17148087	17158306	LCR22-2'
48	19899202	19909416	LCR22-4'
48	23337488	23348064	LCR22-8'
49	17081509	17083237	LCR22-2'
49	18883574	18885303	LCR22-3a'
49	19004460	19006188	LCR22-3a'
49	19835377	19837111	LCR22-4'
49	20024068	20025796	LCR22-4'
50	17041153	17041561	LCR22-2'
50	17124650	17125058	LCR22-2'
50	17232915	17233323	LCR22-2'

50	18729693	18730101	LCR22-3a'
50	18962480	18962888	LCR22-3a'
50	19875683	19876091	LCR22-4'
50	19985636	19986044	LCR22-4'
50	22136551	22136959	LCR22-6'
51	21005158	21012344	LCR22-5'
51	21086889	21094784	LCR22-5'
52	21350334	21351679	LCR22-5'
52	21371980	21373335	LCR22-5'
52	21415308	21416652	LCR22-5'
53	18782103	18845953	LCR22-3a'
53	20063492	20127380	LCR22-4'
53	20176048	20239928	LCR22-4'
54	17144114	17148088	LCR22-2'
54	18939431	18943421	LCR22-3a'
54	19895222	19899203	LCR22-4'
54	23348063	23352022	LCR22-8'
55	17133968	17143534	LCR22-2'
55	18739005	18748581	LCR22-3a'
55	18944001	18953568	LCR22-3a'
55	19884997	19894642	LCR22-4'
55	21318004	21327587	LCR22-5'
55	22967967	22976260	LCR22-7'
55	23352603	23362191	LCR22-8'
56	17038937	17041154	LCR22-2'
56	17125057	17127272	LCR22-2'
56	17230701	17232916	LCR22-2'
56	18730100	18732315	LCR22-3a'
56	18960265	18962481	LCR22-3a'
56	19876090	19878305	LCR22-4'
56	19983422	19985637	LCR22-4'
57	22614950	22615285	LCR22-7'
57	22619372	22619583	LCR22-7'
57	22667201	22667413	LCR22-7'
57	22672089	22672422	LCR22-7'
57	22677434	22677766	LCR22-7'
57	22714165	22714513	LCR22-7'
57	22720053	22720385	LCR22-7'
57	22726020	22726352	LCR22-7'
57	22612605	22612727	LCR22-7'
57	22617594	22617727	LCR22-7'

57	22667751	22667883	LCR22-7'
57	22672755	22672877	LCR22-7'
57	22680279	22680406	LCR22-7'
57	22715037	22715175	LCR22-7'
57	22721004	22721137	LCR22-7'
57	22726991	22727118	LCR22-7'
58	21359721	21367895	LCR22-5'
58	21420384	21428665	LCR22-5'
59	22612728	22612966	LCR22-7'
59	22617728	22617990	LCR22-7'
59	22667884	22668143	LCR22-7'
59	22672878	22673116	LCR22-7'
59	22677767	22678417	LCR22-7'
59	22680407	22680655	LCR22-7'
59	22715176	22715447	LCR22-7'
59	22721138	22721400	LCR22-7'
59	22727119	22727367	LCR22-7'
60	17129556	17131323	LCR22-2'
60	17216777	17218498	LCR22-2'
60	18734601	18736369	LCR22-3a'
60	18956211	18957979	LCR22-3a'
60	19376657	19378428	LCR22-3b'
60	19807875	19809595	LCR22-4'
60	19880589	19882355	LCR22-4'
60	19969501	19971221	LCR22-4'
60	21312459	21314231	LCR22-5'
60	21991522	21993287	LCR22-6'
60	22980312	22982075	LCR22-7'
60	23380638	23382408	LCR22-8'
61	21337576	21339908	LCR22-5'
61	21355919	21358326	LCR22-5'
61	21373577	21375915	LCR22-5'
61	21417054	21419043	LCR22-5'
62	17063114	17064086	LCR22-2'
62	19023700	19024672	LCR22-3a'
63	17255117	17256340	LCR22-2'
63	22155924	22157168	LCR22-6'
64	17037603	17038938	LCR22-2'
64	17127271	17128606	LCR22-2'
64	18732314	18733649	LCR22-3a'
64	18958927	18960266	LCR22-3a'

64	19878304	19879637	LCR22-4'
65	17067993	17068854	LCR22-2'
65	17097452	17098314	LCR22-2'
65	17261286	17262154	LCR22-2'
65	19019022	19019883	LCR22-3a'
66	21351925	21352679	LCR22-5'
66	21416670	21416812	LCR22-5'
67	18775624	18775889	LCR22-3a'
67	19415726	19415976	LCR22-3b'
67	20169400	20169665	LCR22-4'
68	17302263	17303873	LCR22-2'
68	18667209	18668817	LCR22-3a'
69	17227108	17228221	LCR22-2'
69	19798152	19799264	LCR22-4'
69	19979831	19980944	LCR22-4'
69	22990694	22991800	LCR22-7'
70	22613441	22614473	LCR22-7'
70	22618422	22619072	LCR22-7'
70	22666244	22666898	LCR22-7'
70	22670588	22671615	LCR22-7'
70	22675941	22676963	LCR22-7'
70	22712599	22713670	LCR22-7'
70	22718560	22719582	LCR22-7'
70	22724527	22725549	LCR22-7'
70	22681132	22682149	LCR22-7'
70	22727844	22728847	LCR22-7'
71	21368185	21371090	LCR22-5'
71	21405209	21407761	LCR22-5'
71	21429486	21431884	LCR22-5'
71	21463133	21465676	LCR22-5'
71	21493407	21495966	LCR22-5'
72	18776142	18776422	LCR22-3a'
72	19416229	19416448	LCR22-3b'
72	20169919	20170199	LCR22-4'
73	22003973	22005382	LCR22-6'
73	23387942	23389395	LCR22-8'
74	22620456	22620762	LCR22-7'
74	22664982	22665281	LCR22-7'
74	22710940	22711250	LCR22-7'
74	22716903	22717217	LCR22-7'
75	22005381	22009322	LCR22-6'

75	23005649	23009935	LCR22-7'
75	23389394	23393325	LCR22-8'
76	22674594	22674747	LCR22-7'
76	22683276	22683448	LCR22-7'
77	17047526	17047965	LCR22-2'
77	17118278	17118717	LCR22-2'
77	17239292	17239731	LCR22-2'
77	18723294	18723733	LCR22-3a'
77	18968852	18969288	LCR22-3a'
77	19869279	19869718	LCR22-4'
77	19992009	19992448	LCR22-4'
77	22143050	22143190	LCR22-6'
78	21343388	21344444	LCR22-5'
78	21408994	21410030	LCR22-5'
78	21433109	21434360	LCR22-5'
79	17213294	17216295	LCR22-2'
79	19373165	19376170	LCR22-3b'
79	19810076	19813079	LCR22-4'
79	19966028	19969022	LCR22-4'
79	21314712	21317405	LCR22-5'
79	22976824	22979831	LCR22-7'
79	23382890	23385895	LCR22-8'
80	17049170	17059238	LCR22-2'
80	17106814	17116867	LCR22-2'
80	17241175	17251240	LCR22-2'
80	18711788	18721855	LCR22-3a'
80	18971012	18981071	LCR22-3a'
80	19028548	19038632	LCR22-3a'
80	19857506	19867564	LCR22-4'
80	19993812	20003875	LCR22-4'
80	22143354	22152921	LCR22-6'
81	21298342	21300078	LCR22-5'
81	22962831	22963923	LCR22-7'
81	23367401	23369135	LCR22-8'
82	22678416	22678812	LCR22-7'
82	22680253	22680280	LCR22-7'
82	22726964	22726992	LCR22-7'
83	21408660	21408995	LCR22-5'
83	21432779	21433110	LCR22-5'
84	17317753	17335065	LCR22-2'
84	18638726	18657790	LCR22-3a'

85	17042330	17042845	LCR22-2'
85	17123369	17123885	LCR22-2'
85	17234088	17234603	LCR22-2'
85	18728413	18728928	LCR22-3a'
85	18963653	18964168	LCR22-3a'
85	19073926	19074436	LCR22-3a'
85	19874403	19874918	LCR22-4'
85	19986809	19987324	LCR22-4'
85	22137712	22138229	LCR22-6'
86	18756180	18775625	LCR22-3a'
86	19384607	19404072	LCR22-3b'
86	20149943	20169401	LCR22-4'
87	21569549	21573722	LCR22-5'
87	21574897	21579078	LCR22-5'
88	18869809	18874820	LCR22-3a'
88	18990629	18995642	LCR22-3a'
88	20034621	20039631	LCR22-4'
89	22637776	22639775	LCR22-7'
89	22645946	22647946	LCR22-7'
89	22702101	22703811	LCR22-7'
90	19369207	19371306	LCR22-3b'
90	22992108	22994204	LCR22-7'
91	18846090	18852334	LCR22-3a'
91	20057111	20063355	LCR22-4'
91	20240354	20247118	LCR22-4'
92	17143533	17143849	LCR22-2'
92	18943686	18944002	LCR22-3a'
92	19894641	19894957	LCR22-4'
92	21317686	21318005	LCR22-5'
92	22976259	22976577	LCR22-7'
92	23352287	23352604	LCR22-8'
93	17269510	17292230	LCR22-2'
93	18669157	18691077	LCR22-3a'
94	21376652	21382799	LCR22-5'
94	21434410	21440989	LCR22-5'
95	17228220	17230702	LCR22-2'
95	19795675	19798153	LCR22-4'
95	19980943	19983423	LCR22-4'
96	18748703	18756181	LCR22-3a'
96	20142478	20149944	LCR22-4'
97	19356939	19358895	LCR22-3b'

97	20802755	20804973	LCR22-5'
98	22244487	22248921	LCR22-6'
98	22309895	22314095	LCR22-6'
99	17042844	17045788	LCR22-2'
99	17120426	17123370	LCR22-2'
99	17234602	17237555	LCR22-2'
99	18725471	18728414	LCR22-3a'
99	18964167	18967114	LCR22-3a'
99	19871456	19874404	LCR22-4'
99	19987323	19990271	LCR22-4'
99	22138228	22141298	LCR22-6'
100	22673593	22674048	LCR22-7'
100	22682148	22682661	LCR22-7'
100	22616553	22616815	LCR22-7'
100	22668921	22669185	LCR22-7'
100	22715910	22716365	LCR22-7'
100	22721877	22722332	LCR22-7'
101	17223405	17227109	LCR22-2'
101	19799263	19802968	LCR22-4'
101	19976128	19979832	LCR22-4'
101	21303851	21307548	LCR22-5'
101	21982931	21986636	LCR22-6'
101	22986987	22990695	LCR22-7'
101	23372026	23375727	LCR22-8'
102	17077382	17081510	LCR22-2'
102	18692115	18696552	LCR22-3a'
102	18885302	18889428	LCR22-3a'
102	19006187	19010318	LCR22-3a'
102	19837110	19841258	LCR22-4'
102	20019946	20024069	LCR22-4'
103	22009405	22011135	LCR22-6'
103	23012724	23014452	LCR22-7'
103	23393408	23395137	LCR22-8'
104	17161130	17192499	LCR22-2'
104	19912240	19943749	LCR22-4'
105	17131322	17133969	LCR22-2'
105	18736368	18739006	LCR22-3a'
105	18953567	18956212	LCR22-3a'
105	19882354	19884998	LCR22-4'
105	21293084	21295714	LCR22-5'
105	22965328	22967968	LCR22-7'

105	23362190	23365122	LCR22-8'
106	18852333	18853206	LCR22-3a'
106	20056239	20057112	LCR22-4'
107	17083236	17092539	LCR22-2'
107	18874819	18883575	LCR22-3a'
107	18995641	19004461	LCR22-3a'
107	20025795	20034622	LCR22-4'
108	17073198	17077383	LCR22-2'
108	18696551	18700739	LCR22-3a'
108	19010317	19014511	LCR22-3a'
108	19841257	19845450	LCR22-4'
108	20015752	20019947	LCR22-4'
109	17060378	17063115	LCR22-2'
109	17102937	17105674	LCR22-2'
109	17252380	17255118	LCR22-2'
109	18707912	18710649	LCR22-3a'
109	18982210	18984947	LCR22-3a'
109	19024671	19027408	LCR22-3a'
109	19853629	19856366	LCR22-4'
109	20005015	20007752	LCR22-4'
109	22153193	22155925	LCR22-6'
110	18775888	18776143	LCR22-3a'
110	19404071	19404327	LCR22-3b'
110	19415975	19416230	LCR22-3b'
110	20169664	20169920	LCR22-4'
111	19351885	19355628	LCR22-3b'
111	20799047	20802756	LCR22-5'
112	17037138	17037604	LCR22-2'
112	17128605	17129062	LCR22-2'
112	18733648	18734106	LCR22-3a'
112	18958471	18958928	LCR22-3a'
112	19046917	19047399	LCR22-3a'
112	19879636	19880093	LCR22-4'
113	18776497	18782104	LCR22-3a'
113	19416627	19422338	LCR22-3b'
113	20170475	20176049	LCR22-4'
114	17045787	17047419	LCR22-2'
114	17118824	17120427	LCR22-2'
114	17237554	17239185	LCR22-2'
114	18723840	18725472	LCR22-3a'
114	18967113	18968745	LCR22-3a'

114	19869825	19871457	LCR22-4'
114	19990270	19991902	LCR22-4'
114	22141482	22143051	LCR22-6'
115	21296104	21296574	LCR22-5'
115	21297746	21298223	LCR22-5'
115	22964041	22964516	LCR22-7'
115	23365495	23365665	LCR22-8'
115	23366808	23367282	LCR22-8'
116	17158305	17161034	LCR22-2'
116	19909415	19912144	LCR22-4'
116	22342419	22343104	LCR22-6'
116	23334750	23337489	LCR22-8'
117	17022490	17037082	LCR22-2'
117	19047455	19061985	LCR22-3a'
118	23026663	23027095	LCR22-7'
118	23404382	23404814	LCR22-8'
119	19362636	19368020	LCR22-3b'
119	19741670	19747026	LCR22-4'
120	17048030	17048595	LCR22-2'
120	17117649	17118213	LCR22-2'
120	17239796	17240357	LCR22-2'
120	18722664	18723229	LCR22-3a'
120	18969353	18969915	LCR22-3a'
120	19046356	19046918	LCR22-3a'
120	19868652	19869214	LCR22-4'
120	19992513	19993075	LCR22-4'
120	22143189	22143288	LCR22-6'
121	22675802	22675942	LCR22-7'
121	22684640	22684799	LCR22-7'
121	22613300	22613442	LCR22-7'
121	22618333	22618422	LCR22-7'
121	22666155	22666245	LCR22-7'
121	22670448	22670589	LCR22-7'
121	22712453	22712600	LCR22-7'
121	22718420	22718561	LCR22-7'
121	22724387	22724528	LCR22-7'
122	22002884	22003182	LCR22-6'
122	23005043	23005342	LCR22-7'
