# Supplementary material for: Characterization of the past and current duplication activities in the human 22q11.2 region
Source: BMC Genomics. 2011 Jan 26;12:71. doi: 10.1186/1471-2164-12-71 (PMC3040729; doi:10.1186/1471-2164-12-71)

Additional File 3.

Supplementary Figure S2. PCR Analysis Confirmed a Duplication Event Absent in the Macaque Genome.

We carried out PCR analysis for a duplicated sequence (chr22:21,293,079-21,327,588; hg18) that was predicted to be specific to the human and chimp genomes from our sytenic analysis. PCR was performed using the FastStart High Fidelity PCR System (Roche 03553361001). Each 25µl PCR reaction contains 1X PCR Buffer, 10% DMSO, 3mM $MgCl_2$, 0.2mM dNTPs, 0.5µM forward primer, 0.5µM reverse primer, 0.25µl Taq polymerase, 120ng DNA. PCR products were loaded with 10% glycerol and run on a 2% agarose gel containing 0.3mg/mL ethidium bromide. Due to different intensities of products, PCR products in lanes 3 and 4 were diluted 1:2 and PCR products in lanes 5 and 6 were diluted 1:7 prior to loading on gel. Macaque DNA was isolated from ells grown from clone AG07109 (Coriell). 60ng of each PCR product together with 3.2pmol primer was submitted for traditional Sanger sequencing performed by the Albert Einstein College of Medicine Genomics Core Facility. The PCR data and sequencing result supported that the duplication is absent in macaques.

A. Primer sequences and positions:

2-1F: ATATGAAGGATGACTCCCTAGG
2-1R: AGATATTAAGATAACCATTAGAACA
2-2F: AAGGAACATGGTGACAGTGAG
2-2R: GGGTCAGCAAACCATAGTGA

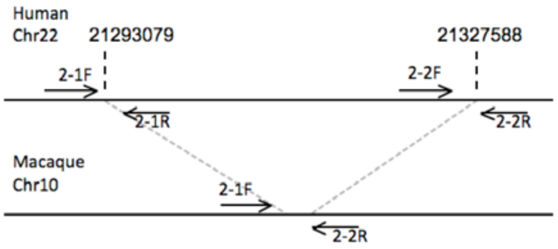

B. PCR product:

Lane	Genomic DNA	Primers	PCR Product Size (bp)
1	Human	2-1F + 2-1R	334
2	Macaque	2-1F + 2-1R	0
3	Human	2-2F + 2-2R	852
4	Macaque	2-2F + 2-2R	0
5	Human	2-1F + 2-2R	0
6	Macaque	2-1F + 2-2R	793

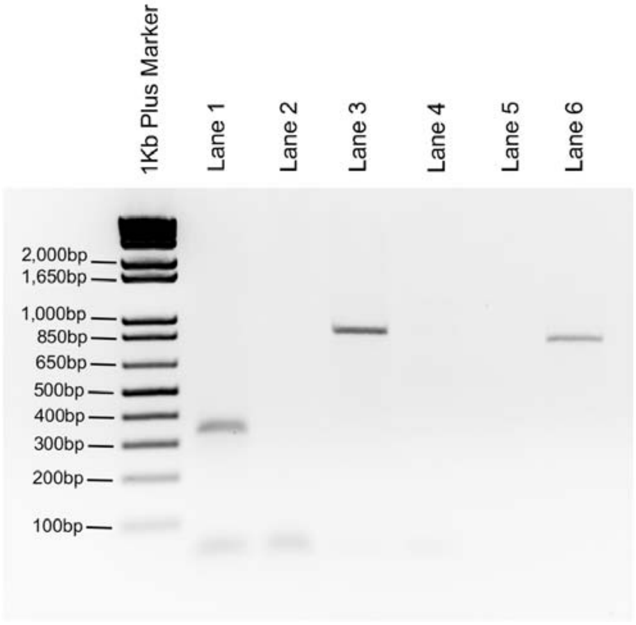

Supplement: Additional file 3 — Supplementary Figure S2. PCR Analysis Confirmed a Duplication Event Absent in the Macaque Genome. We carried out PCR analysis for a duplicated sequence (chr22:21,293,079-21,327,588; hg18) that was predicted to be specific to the human and chimp genomes from our sytenic analysis. [file 1471-2164-12-71-S3.PDF]
