# Supplementary material for: Characterization of the past and current duplication activities in the human 22q11.2 region
Source: BMC Genomics. 2011 Jan 26;12:71. doi: 10.1186/1471-2164-12-71 (PMC3040729; doi:10.1186/1471-2164-12-71)
Supplement: Additional file 4 — Supplementary Figure S3. Comparison of Currently Defined SD Subunits with those Defined by Jiang et al. in Previous Work [ref [21] and ref [23]]. [file 1471-2164-12-71-S4.PDF]

Additional File 4.

Supplementary Figure S3. Comparison of Currently Defined SD Subunits with those Defined by Jiang et al. in Previous Work [ref 21 and ref 23].

In our current work, the 22q11.2 segmental duplications (SDs) were decomposed into 502 subunits. Previously, Jiang *et al* has also applied the *A-Bruijin* graph algorithm to decompose all human SDs (hg17 version) into core duplication subunits [ref 21 and ref 23]; 1003 of those subunits were in 22q11.2. Figure A illustrates the comparison of these two independently defined sets of subunits. The colors for current subunits depict paralogous relationships as in Figure 2 of the main text, while the colors for Jiang *et al*'s subunits are simply used to separate adjacent subunits. As shown here, the subunits from Jiang's genome-wide analysis were much smaller than ours that were inferred with SDs in 22q11.2 only. Their average size was 1,565 bp, which is significantly smaller than that (3,240 bp) of our subunits. This is fully expected because inclusion of SDs outside 22q11.2 would further break our defined subunits into smaller sub-sequences. Many subunits in LCR22-6's were present only in Jiang *et al*'s data, suggesting that the underlying SDs did not involve sequences in other LCR22 duplicated blocks and thus they may not contribute to intra-LCR22 rearrangements. 69 subunits were defined specifically in our analysis, which might result from our usage of data on a newer assembly of the human genome (hg18). As shown in B, for the common subunits present in both studies, ~70% of their breakpoints defined in our analysis were < 50 bp away from the corresponding breakpoints defined by Jiang *et al*, and this was up to 84% upon increasing of the distance to <200 bp. Interestingly, 33 of the 36 ancestral core subunits defined by Jiang *et al* were also found to be present in other primate genomes in our syntenic analysis.

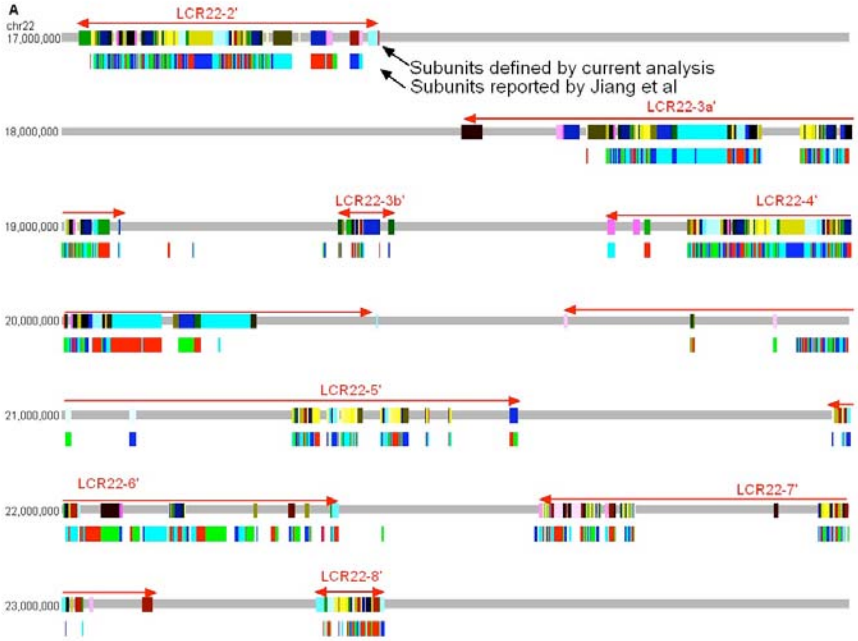
A
chr22
17,000,000
LCR22-2'
Subunits defined by current analysis
Subunits reported by Jiang et al
18,000,000
LCR22-3a'
19,000,000
LCR22-3b'
LCR22-4'
20,000,000
21,000,000
LCR22-5'
22,000,000
LCR22-6'
LCR22-7'
23,000,000
LCR22-8'

B.

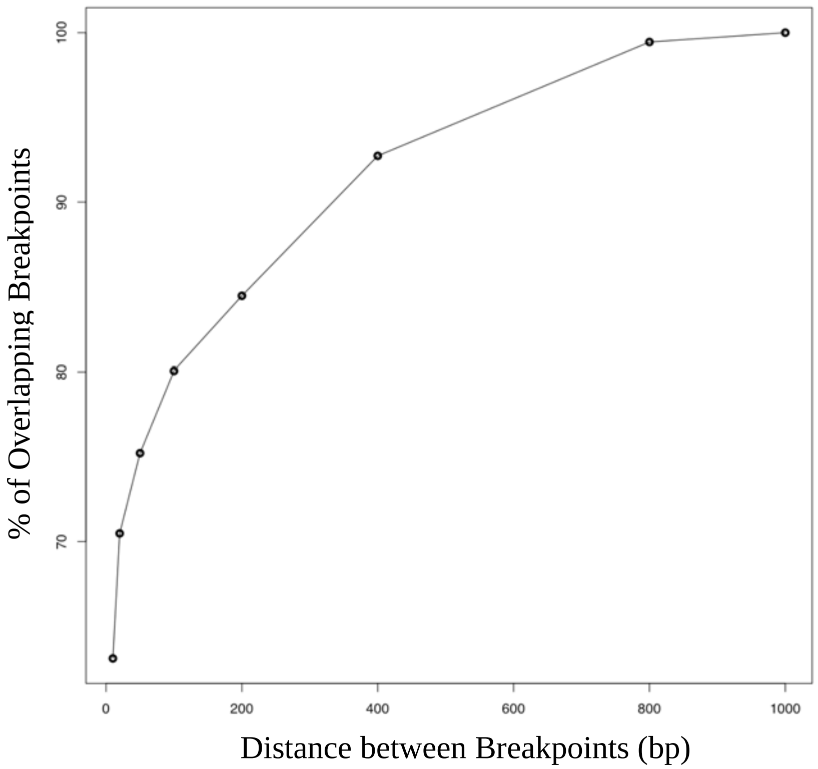
% of Overlapping Breakpoints
70
80
90
100
0
200
400
600
800
1000
Distance between Breakpoints (bp)
